# PRMT6 activates cyclin D1 expression in conjunction with the transcription factor LEF1

**DOI:** 10.1038/s41389-021-00332-z

**Published:** 2021-05-17

**Authors:** Lucas Schneider, Stefanie Herkt, Lei Wang, Christine Feld, Josephine Wesely, Olga N. Kuvardina, Annekarin Meyer, Thomas Oellerich, Björn Häupl, Erhard Seifried, Halvard Bonig, Joern Lausen

**Affiliations:** 1 Goethe University, Institute for Transfusion Medicine and Immunohematology, and German Red Cross Blood Service BaWüHe, Institute Frankfurt, Frankfurt, Germany; 2 Department of Eukaryotic Genetics, Institute of Industrial Genetics, University of Stuttgart, Stuttgart, Germany; 3 Georg-Speyer-Haus, Institute for Tumor Biology and Experimental Therapy, Frankfurt am Main, Germany; 4 Department of Medicine II, Hematology/Oncology, Goethe University, Frankfurt, Germany; 5 German Cancer Research Center and German Cancer Consortium, Heidelberg, Germany; 6 Department of Molecular Diagnostics/Translational Proteomics, Frankfurt Cancer Institute, Frankfurt, Germany; 7 Department of Medicine, Division of Hematology, University of Washington, Seattle, WA USA; 8 Present Address: Automated Systems and Genomics, The New York Stem Cell Foundation Research Institute, New York, USA

**Keywords:** Oncogenes, Cell growth, Cell growth

## Abstract

The establishment of cell type specific gene expression by transcription factors and their epigenetic cofactors is central for cell fate decisions. Protein arginine methyltransferase 6 (PRMT6) is an epigenetic regulator of gene expression mainly through methylating arginines at histone H3. This way it influences cellular differentiation and proliferation. PRMT6 lacks DNA-binding capability but is recruited by transcription factors to regulate gene expression. However, currently only a limited number of transcription factors have been identified, which facilitate recruitment of PRMT6 to key cell cycle related target genes. Here, we show that LEF1 contributes to the recruitment of PRMT6 to the central cell cycle regulator *CCND1* (Cyclin D1). We identified LEF1 as an interaction partner of PRMT6. Knockdown of LEF1 or PRMT6 reduces *CCND1* expression. This is in line with our observation that knockdown of PRMT6 increases the number of cells in G1 phase of the cell cycle and decreases proliferation. These results improve the understanding of PRMT6 activity in cell cycle regulation. We expect that these insights will foster the rational development and usage of specific PRMT6 inhibitors for cancer therapy.

## Introduction

Methylation of arginine residues in histone and non-histone proteins is catalyzed by protein arginine methyltransferases (PRMTs)^1^, which constitute a family of conserved enzymes in mammals. Arginine methylation of histone tails can act repressive or activating on transcription, depending on the specific methylated residue. Furthermore, the functional outcome depends whether asymmetric or symmetric dimethylation has occurred^1–3^. In concert with other histone modifications arginine methylation influences chromatin states, and this way provides epigenetic information. In addition, PRMTs are able to methylate non-histone proteins. In both cases arginine methylation is in crosstalk with other posttranslational modifications, such as lysine methylation or phosphorylation^2^. PRMTs possess no direct DNA binding capability, instead they are recruited to target genes by transcription factors, and can be part of multicomponent transcriptional complexes. As regulators of gene expression and epigenetic information they play an important role in diverse biological processes, such as stem cell functions, proliferation control, and differentiation^1^.

Protein arginine methyltransferase 6 (PRMT6) is a nuclear protein, which asymmetrically dimethylates arginine residues. In the nucleus, PRMT6 predominantly mediates H3R2me2a, a histone modification mark, which represses gene expression by counteracting H3K4me3^4–7^. In this way, PRMT6 also contributes to the presence of bivalent chromatin marking^8,9^. Recently, it was demonstrated that H3R2me2a can have activating function at enhancer regions^10^. Furthermore, PRMT6 is connected to DNA-methylation^11^. Although PRMT6 is mostly described as a transcriptional repressor it also fulfills context dependent gene activating functions^10,12–14^ and influences splicing^15,16^. Furthermore, arginine methylation of non-histone proteins involved in cellular signaling by PRMT6 was described^16–18^.

PRMT6 is present at regulatory DNA regions of important cell cycle regulators such as *CDKN1A*, *CDKN1B*, *CDKN2A*, and *p53*, where it acts as transcriptional repressor. In line with this observation loss of PRMT6 inhibits the cell cycle and increases senescence^19–23^. Furthermore, PRMT6 influences embryonic stem cell identity^24^. In the hematopoietic system the transcription factor RUNX1 recruits PRMT6, which contributes to the repression of RUNX1 erythroid target genes^25–27^. PRMT6 expression is associated with several cancer types^15,28–30^ and pharmacological inhibitors are under investigation as therapeutic inhibitors^31,32^.

The biological outcome of the transcriptional activity of PRMT6 depends on the transcription factors it interacts with. In MCF7 breast cancer cells PRMT6 is associated with the polycomb complex^19^ and interacts with the estrogen receptor α^33^ and the androgen receptor in context of muscular atrophy^34^. Furthermore, PRMT6 coactivates the progesterone, glucocorticoid, and estrogen receptors^12^. Additional, PRMT6 is recruited to target genes by PPARy in adipocyte differentiation^35^ and is associated with NF-kB^14^. Recently, we found that PRMT6 is recruited by RUNX1 during hematopoietic differentiation^8,36^.

In our ongoing effort to understand the connection of PRMT6 and transcription factor activity in cell cycle regulation, we undertook this study to identify interacting transcription factors of PRMT6. We initiated a mass spectrometry screen for nuclear PRMT6 interaction partners and identified LEF1 as interactor of PRMT6. Our data show that LEF1 and PRMT6 cooperate in regulation of the cell cycle gene *CCND1* (Cyclin D1).

## Results

**Fig. 1:**
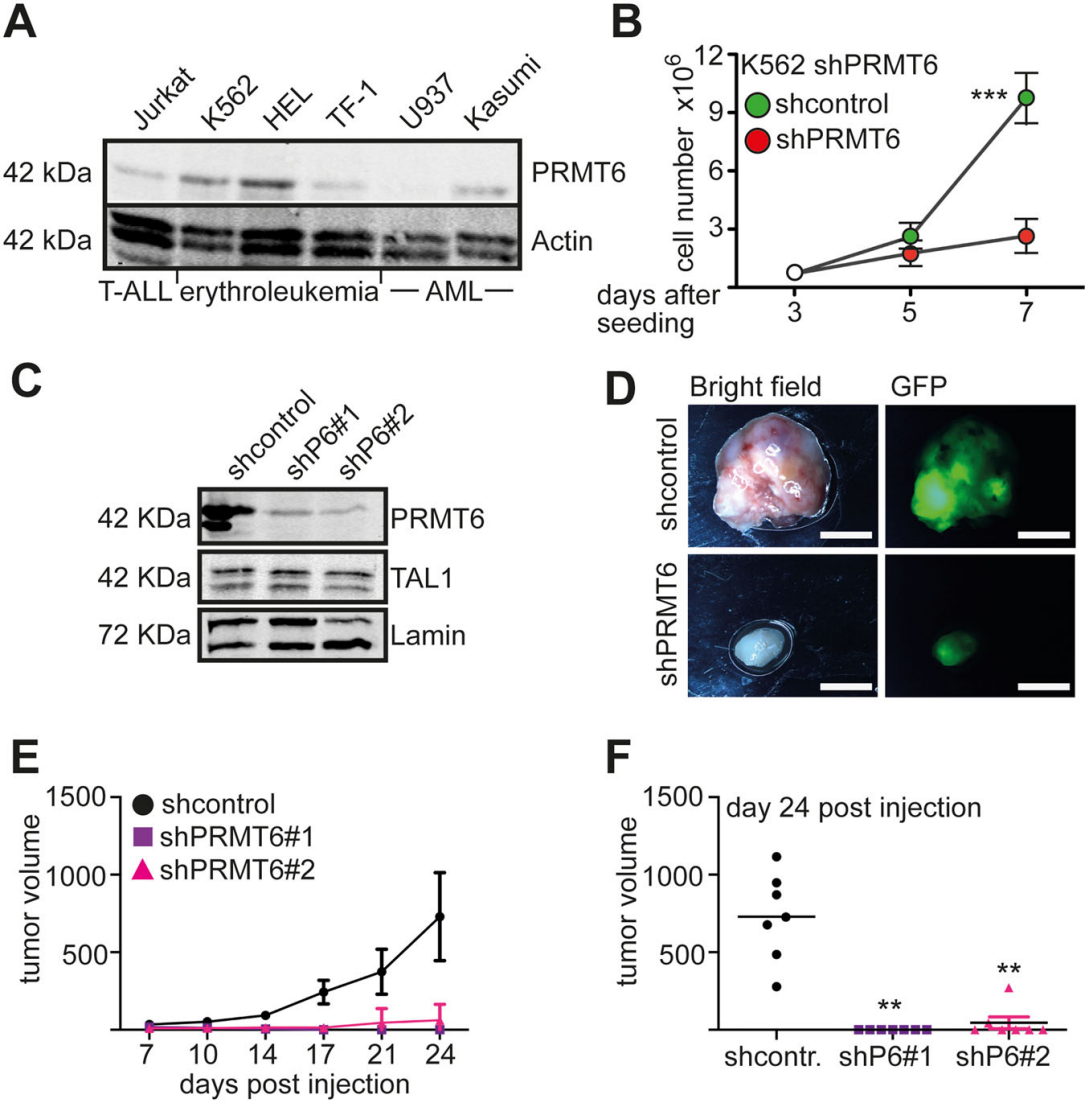
PRMT6 knockdown decreases proliferation of hematopoietic cells lines. **A** Western blot analysis of PRMT6 expression in Jurkat, K562, HEL, TF-1, U937, and Kasumi cells. Western blot was done with extracts from the indicated cells and specific antibodies against PRMT6. Lamin served as loading control. **B** PRMT6 mediates enhanced proliferation. PRMT6 was knocked down by shRNA in K562 cells. Six days after transduction shPRMT6 and shcontrol cells were seeded out in similar numbers. Cells were counted at the indicated time points. The error bars display the standard deviation from the mean from three determinations. The *P*-values were calculated using ANOVA. ***P < 0.001. **C** Western blot showing efficient knockdown of PRMT6 with shRNA. Western blot against the transcription factor TAL1 and against actin served as controls. These cells were injected subcutaneously into C57BL/6 mice. **D** Analysis of subcutaneous tumors upon injection of shcontrol and shPRMT6 K562 cells, respectively. Bright field and GFP image of an exemplary tumor from day 24 is displayed. The white bar indicates 0.5 cm. **E** Tumor growth curve from day 7 after injection until day 24 is shown for two shPRMT6 constructs. Tumor volume is given in mm^3^. **F** Endpoint analysis at day 24 of post-injection. Tumor volume is given. The *P*-values were calculated using ANOVA from seven mice. **P < 0.01, ***P < 0.001.

PRMT6 expression is associated with a number of cancers^28^ and altered cell growth and differentiation. However, little is known about the influence of PRMT6 on proliferation in hematopoiesis. We found that PRMT6 inhibits erythropoiesis and recent data supports the idea that PRMT6 plays a role in cell growth^25^. To further investigate this notion, we determined expression of PRMT6 in distinct hematopoietic cell lines. PRMT6 protein is expressed in the T-ALL cell line Jurkat, in the erythroleukemia cell lines K562, HEL, and TF-1 as well as the AML cell line U937 and Kasumi, with the lowest expression in U937 cells (Fig. 1A).

To analyze the influence of PRMT6 on growth we performed shRNA mediated depletion of PRMT6 in K562 cells. The shPRMT6 transduced K562 cells showed significant slower proliferation than the shcontrol transduced K562 cells (Fig. 1B). To verify this result in a semi in-vivo situation, a mouse tumor model was established. We knocked down PRMT6 with two distinct shRNAs in K562 cells (Fig. 1C) and injected the cells subcutaneously in C57BL/6 mice. Subsequently, tumor growth was monitored during time. The expression of the shPRMT6 as well as the shcontrol was confirmed until the end of the experiment, as the GFP marker was detected until day 24 (Fig. 1D). Whereas the control cells grew to visible tumors, PRMT6 knockdown resulted in small, hardly palpable tumors (Fig. 1D–F). These results support the hypothesis that loss of PRMT6 inhibits cell proliferation in hematopoietic cells similar to results gathered in the breast cancer cell line MCF7^21^ and U2OS osteosarcoma cells^20^.

### Identification of PRMT6 interaction partners

**Fig. 2:**
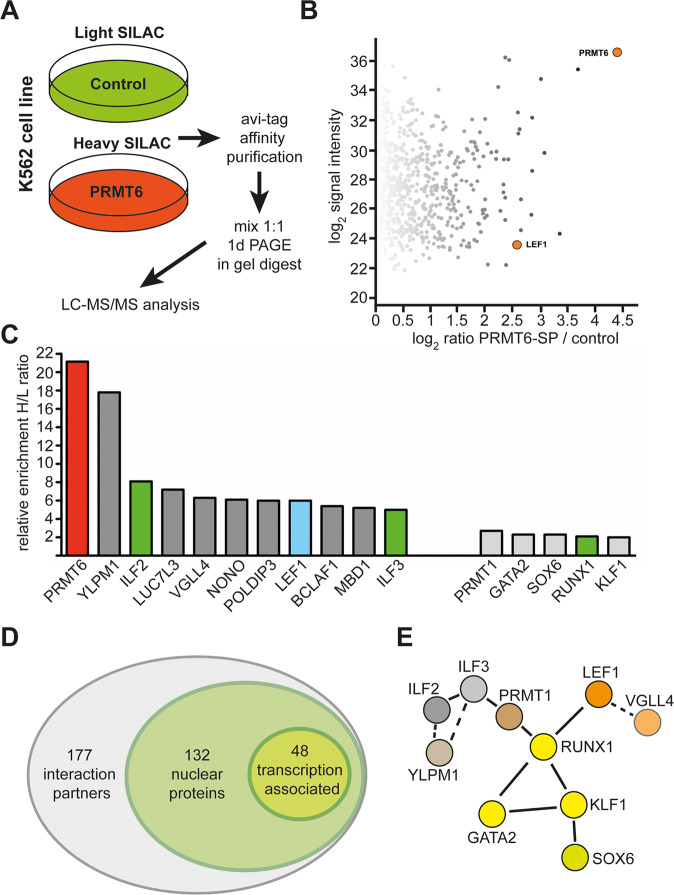
SILAC-based mass spectrometry reveals PRMT6 interactome. **A** Strategy for the identification of PRMT6 interaction partners by SILAC based mass spectrometry upon avi-tag affinity purification of PRMT6. K562 cells were transduced with PRMT6 and the corresponding empty pRRLs-Avi vector control. The control cells were cultured for seven passages in light SILAC medium, containing normal amino acids, avi-PRMT6 over expressing cells were cultured in heavy SILAC medium (amino acids labeled with heavy isotopes). Nuclear extracts were prepared, and avi-tag affinity purification was performed with streptavidin beads. The extract form light and heavy SILAC labeled cells were mixed in a 1:1 ratio and applied to LC-MS/MS analysis. **B** Scatter plot of signaling intensity vs. PRMT6-SP/control. PRMT6 and LEF1 are highlighted in orange. **C** Selected candidate interaction partners of PRMT6. Known interaction partners are marked in green. Relative enrichment of proteins in the bio-PRMT6 sample was determined by calculating the ratio between peak intensities of identified peptides from the heavy (H, bio-PRMT6 + BirA-ligase) versus the light (L, BirA-ligase) sample. The cut-off was enrichment with an H/L ratio of two (which is KLF1). **D** Euler diagram of PRMT6 interactome. Functional annotation clustering with DAVID^8,9^ revealed that most interacting proteins are nuclear located and 48 are associated with transcription. **E** Protein interaction network of transcription factors and associated cofactors revealed by STRING^10^.

Although the detailed mechanism of PRMT6 activity on proliferation in cancer is not yet identified, the recruitment of PRMT6 by transcription factors to target genes involved in cell-cycle regulation is part of this process. To identify transcription factors capable of recruiting PRMT6 to target genes, we used affinity purification of avi-tagged PRMT6 in combination with stable isotope labeling of amino acids in cell culture (SILAC) based mass spectrometry^37^. For this, K562 cells were transduced with the BirA-ligase and avi-PRMT6 with a 21 amino acid tag, which is biotinylated in the cells^38^. Cells expressing only the BirA-ligase served as control. Avi-PRMT6 cells were grown in heavy SILAC medium and control cells in light SILAC medium for seven passages (Fig. 2A). Subsequently, we performed affinity purification and mass spectrometry. The relative enrichment of proteins in the avi-PRMT6 cells was determined by calculating the ratio between peak intensities of identified peptides from the heavy (H, avi-PRMT6 + BirA-ligase) versus the light (L, BirA-ligase) sample (Fig. 2B, C). Besides PRMT6 itself, we identified several known PRMT6 interaction partners such as ILF2, ILF3, PRMT1, and RUNX1, thus validating the assay (Fig. 2C). Potential interaction partners with an H/L ratio of more than five are involved in RNA-binding and splicing (ILF2, ILF3, LUC7L3, and NONO) or in gene expression regulation (YLPM1, VGLL4, POLDIP3, LEF1, BCLAF1, and MBD1). Of these BCLAF1 and LEF1 are DNA binding transcription factors^39–41^. Altogether 177 putative interacting proteins were identified, 132 of these were nuclear proteins and 48 were associated with transcription (Fig. 2D and Supplementary Table 1). We performed STRING (protein–protein interaction networks functional enrichment analysis^42^) analysis of the potential interaction partners (Fig. 2E). These associated proteins potentially constitute a PRMT6-related interaction network. Of those, VGLL4, RUNX1, and LEF1 are connected to the growth regulating wnt/β-catenin pathway^43–45^. Taken together, we identified novel PRMT6 interaction partners. We decided to further analyze the LEF1/PRMT6 connection, because LEF1 could recruit PRMT6 to specific target genes important for the growth regulating function of LEF1^39,43^.

### Interaction of PRMT6 with LEF1

LEF1 binds to binding sites in promoters and is a mediator of WNT-signaling as it is associated with β-Catenin in case of an activated β-Catenin signaling^39^. In this case LEF1 acts as an activator of its target genes. In the absence of β-catenin it serves as a repressor^46^.

**Fig. 3:**
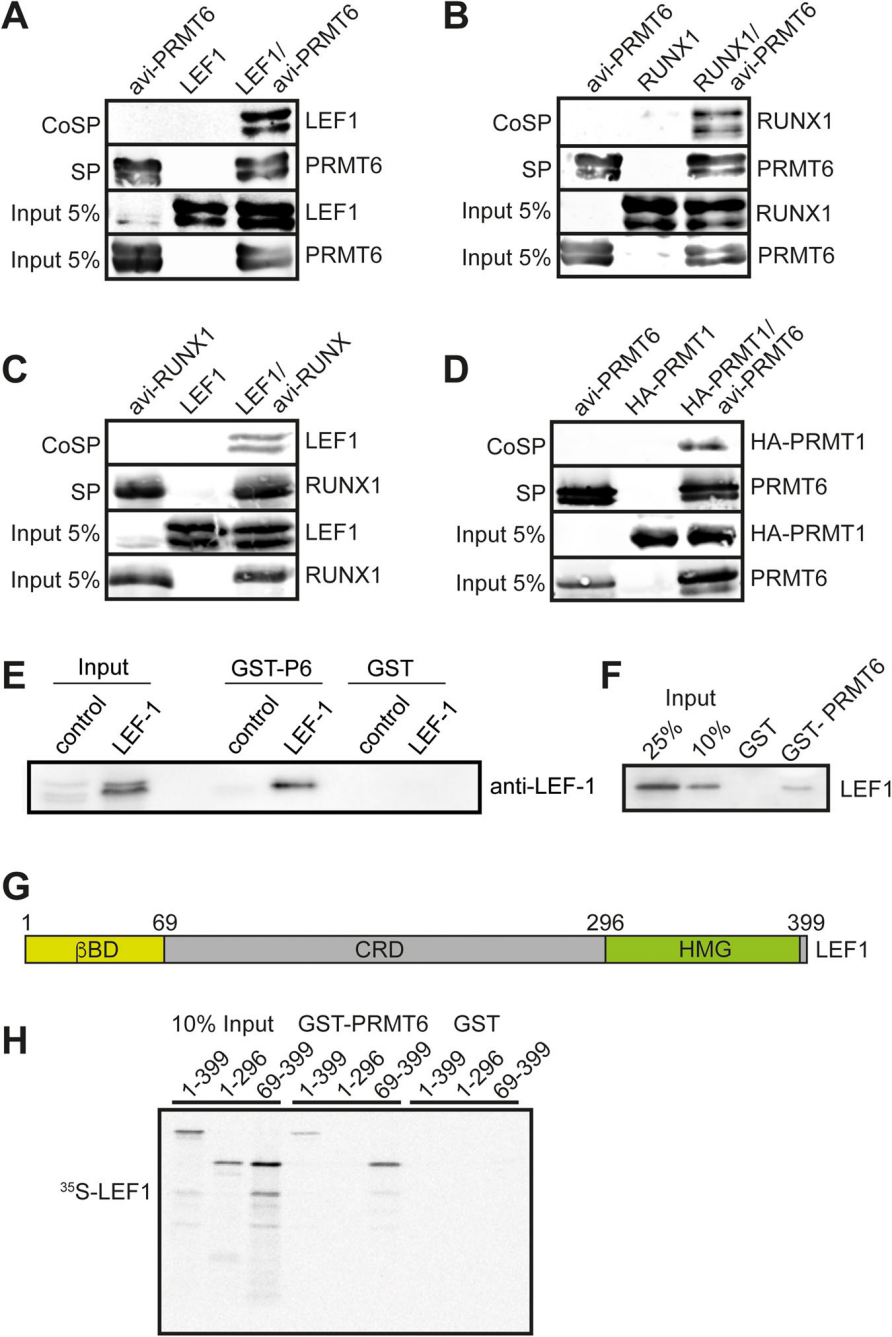
LEF1 is a novel identified PRMT6 interaction partner. Co-streptavidin-precipitation (CoSP). The biotin tagged bait was co-transfected with the prey expression vector into HEK293 cells. The biotinylated bait was pulled out from extracts with streptavidin beads (SP). Coprecipitated bait protein is shown in the upper lane, (CoSP). **A** CoSP of PRMT6 and LEF1. Coprecipitation of LEF1 was detected with an anti-LEF1 antibody. **B** CoSP of PRMT6 and RUNX1. Coprecipitation of RUNX1 was detected with an anti-RUNX1 antibody**. C** CoSP of RUNX1 and LEF1. Biotinylated RUNX1 protein was pulled out from extracts with streptavidin beads. Coprecipitation of LEF1 was detected with an anti-LEF1 antibody. **D** CoSP of PRMT6 and PRMT1. Coprecipitation of PRMT1 was detected with an anti-HA antibody. **E** GST-pulldown with GST-PRMT6 as bait and LEF1 protein expressed in HEK293 cells. GST-PRMT6 was incubated with cell extracts of from LEF1 over expressing cells. GST-pulldown with GST protein served as negative control. The GST proteins were pulled out with glutathione beads. Pulled out LEF1 was detected by western blot with anti-LEF1 antibody. **F** GST-pulldown with GST-PRMT6 and in vitro translated LEF1. Pulled out LEF1 was detected by western blot with anti-LEF1 antibody. **G** Schematic representation of the LEF1 protein. **H** GST-pulldown with GST-PRMT6 and in vitro translated ^35^S labeled LEF1 deletion constructs. GST protein served as negative control and the GST proteins were pulled out with glutathione beads. Detection of pulled out LEF1 protein was done by radiography.

To verify the association of PRMT6 with LEF1 we performed co-streptavidin-precipitations (CoSP) in HEK293 cells. In this case avi-PRMT6 was transfected with LEF1 and CoSP was done with streptavidin-beads, which bind the biotin tagged protein. LEF1 and PRMT6 robustly copurified in this setting (Fig. 3A), the known interaction of PRMT6 and RUNX1 served as a positive control^8,36^ (Fig. 3B). We could also confirm the interaction of RUNX1 with LEF1^47^ (Fig. 3C) and the association of PRMT6 with PRMT1^48^ (Fig. 3D).

To further characterize the interaction between LEF1 and PRMT6, we performed independent assays. For this, we expressed and purified PRMT6 as GST-fusion protein in *E. coli* and prepared extracts from LEF1 over expressing HEK293 cells. In a GST pull-down GST-PRMT6 interacted with LEF1 from cell extracts (Fig. 3E). Similarly, GST-PRMT6 interacted with LEF1 from an in vitro transcription translation reaction (Fig. 3F). These GST pull down assays verified the interaction of PRMT6 with LEF1. LEF1 has several functional domains (Fig. 3G). We mapped the interaction domain of LEF1 with PRMT6 by GST pull-down with GST-PRMT6 and S^35^-labeled in vitro translated LEF1 deletion constructs (Fig. 3H and Supplementary Fig. 1). Deletion of the β-catenin binding site located at amino acids 1–69 of LEF1^39^ did not interrupt binding to PRMT6. However, deletion of the HMG domain at the C-terminus of LEF1 resulted in loss PRMT6 interaction. The HMG domain is the DNA-binding domain of LEF1^49^.

In summary, we verified the interaction between LEF1 and PRMT6 and located the interaction to the HMG-domain of LEF1. Therefore, we reasoned that LEF1 has the potential to mediate PRMT6 recruitment to chromatin.

### Identification of CCND1 as a cell cycle relevant PRMT6/LEF1 target gene

**Fig. 4:**
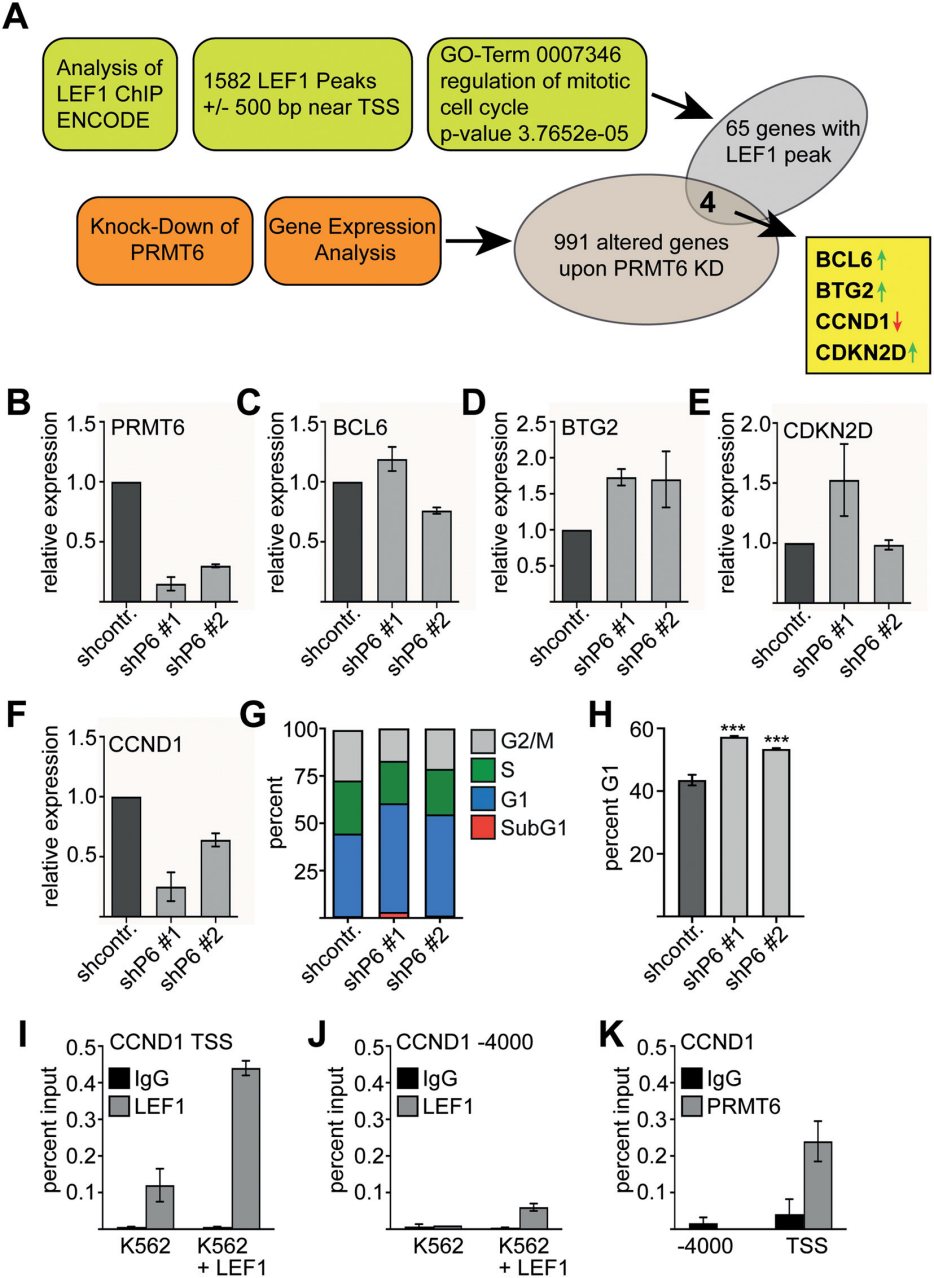
Combined ChIP-seq and RNA transcriptome analysis reveals *CCND1* as a direct LEF1/PRMT6 target. **A** Evaluation of LEF1 ChIP Encode data in K562 cells and GO-term analysis revealed 65 genes regulating the mitotic cell cycle, which are bound by LEF1. Expression analysis identified 991 differentially expressed genes upon knockdown of PRMT6 in K562 cells. Of these, four genes are cell cycle associated LEF1 targets, BCL6, BTG2, CCND1, and CDKN2D. The arrows indicate upregulation or downregulation upon PRMT6 knockdown. **B** mRNA expression analysis of two different shRNA constructs against PRMT6 (shP6). GAPDH expression was used for normalization. **C**–**F** The four identified cell cycle associated LEF1/PRMT6 targets were re-analysed by quantitative real-time PCR 7 days after shPRMT6 transduction. Error bars represent the standard deviation from at least three independent experiments. **G** Cell cycle analysis was performed five days after PRMT6 knockdown in K562 cells. **H** Percentage of cells within the G1 phase of the cell cycle increased upon PRMT6 knockdown in K562 cells. **I**, **J** ChIP assay shows that LEF1 is bound close to the *CCND1* transcriptional start site (TSS). This binding is increased upon LEF1 over expression. The negative control (−4000 bp from the TSS) is displayed in **J**. **K** ChIP-assay shows that PRMT6 is bound close to the *CCND1* transcriptional start site (TSS), but not to the −4000 region. ChIP were performed with an anti-LEF1 and anti-PRMT6 antibody, respectively. The P-values were calculated using Student’s *t*-test from at least three independent measurements. *P < 0.05; **P < 0.01; ***P < 0.001.

To examine the notion that LEF1 might be able to mediate PRMT6 recruitment to LEF1 target genes, we wanted to identify common LEF1/PRMT6 target genes. PRMT6 influences cell cycle associated genes such as *CDKN1A*, *CDKN1B*, *CDKN2A*, and *p53*^19–23^. Furthermore, the GO-term regulation-of-cell-proliferation was enriched in our list of genes, which were differentially expressed upon knockdown of PRMT6 in K562 cells^25^ (Supplementary Fig. 2). Thus, we focused on potential common LEF1/PRMT6 target genes, which were associated with the cell-cycle. To this aim, we analyzed ChIP-sequencing data of LEF1 in K562 cells deposited to Encode^50,51^. We identified LEF1 peaks close to the transcriptional start site (TSS) of 1582 genes (Supplementary Fig. 2 and Supplementary Table 2). To derive potential LEF1/PRMT6 target genes involved in cell cycle regulation, we performed gene ontology analysis. Sixty-five potential LEF1 target genes with an involvement in cell cycle regulation were identified (Fig. 4A and Supplementary Table 2). We finally derived a list of four cell cycle genes by overlapping the previous set of genes, which were differentially expressed upon knockdown of PRMT6 in K562 cells^25^. These criteria led to the identification of four cell cycle associated genes with a LEF1/PRMT6 connection (Supplementary Fig. 3). *BCL6* (B-cell lymphoma 6), *BTG2* (BTG family member 2) and *CDKN2D* (Cyclin Dependent Kinase Inhibitor 2D) were upregulated upon PRMT6 knockdown, whereas *CCND1* (Cyclin 69D1) expression decreased upon PRMT6 knockdown (Fig. 4A).

To probe the connection of those four genes with LEF/PRMT6, we independently knocked down PRMT6 in K562 cells (Fig. 4B) and examined *BCL6*, *BTG2, CDKN2D* and *CCND1* expression. *BCL6* was not changed upon PRMT6 knockdown in this experiment (Fig. 4C). *BTG2* expression increased upon down regulation of PRMT6 (Fig. 4D). The results for *CDKN2D* remained inconclusive (Fig. 4E), whereas *CCND1* expression was reduced (Fig. 4F). *CCND1* encodes for cyclin D1, a central regulator of the cell cycle and a prominent oncogene^52^. Furthermore, CCND1 is a target for activation by β-Catenin^53^. We therefore analyzed the cell cycle upon PRMT6 knockdown. We found an increased number of cells within the G1 phase of the cell cycle upon PRMT6 knockdown in K562 cells (Fig. 4G, H and Supplementary Fig. 4). The cells in the sub-G1 area remained unchanged, indicating that apoptosis was not altered significantly.

We further examined the connection of PRMT6/LEF1 with *CCND1* expression. Chromatin immunoprecipitation (ChIP) revealed that LEF1 binds close to the transcriptional start site (TSS) of *CCND1* (Fig. 4I). Over expression of LEF1 increased LEF1 binding to this site (Fig. 4I), a region −4000 of the *CCND1* start site served as negative control (Fig. 4J). Furthermore, we detected PRMT6 binding close to the TSS of *CCND1* (Fig. 4K). Taken together, our data show that *CCND1* is a direct target gene of LEF1 and PRMT6 and contributes to a block of G1 phase exit.

### LEF1 regulates cyclin D1 and BCL6 expression in K562 cells

**Fig. 5:**
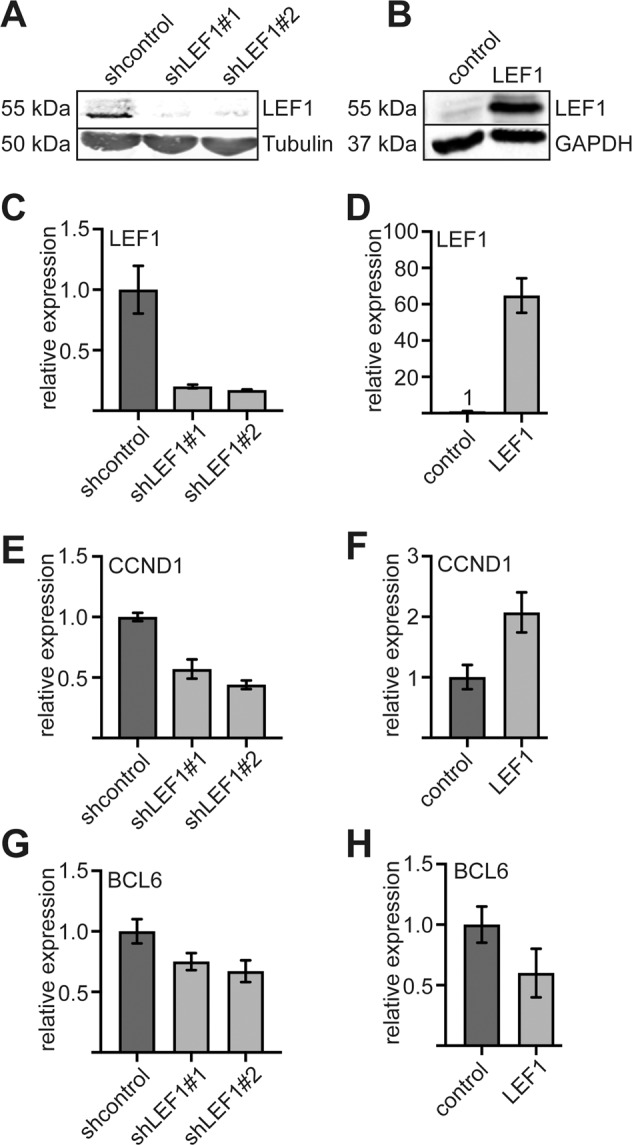
LEF1 knockdown and over expression in K562 cells confirms influence of LEF1 on *CCND1* expression. **A** Western blot analysis of LEF1 knockdown in K562 cells. **B** Western blot analysis of LEF1 over expression in K562 cells. **C** mRNA level of LEF1 is decreased in LEF1 knockdown cells**. D** mRNA level of LEF1 is increased in LEF1 over expression cells. **E** *CCND1* mRNA expression decreased after LEF1 knockdown. **F** mRNA level of *CCND1* after LEF1 over expression in K562 cells. **G** BCL6 mRNA expression decreased after LEF1 knockdown. **H** mRNA level of BCL6 after LEF1 over expression in K562 cells. Error bars represent the standard deviation from the mean from at least three independent determinations. The *P*-values were calculated using Student’s *t*-test. *P < 0.05; **P < 0.01; ***P < 0.001.

To examine the influence of LEF1 on cyclin D1 expression in K562 cells we knocked down LEF1 by two shLEF1 constructs (Fig. 5A, C) and over expressed LEF1 (Fig. 5B, D). Knockdown of LEF1 led to decreased *CCND1* expression (Fig. 5E) and over expression of LEF1 increased *CCND1* expression (Fig. 5F). BCL6 is also a LEF1/PRMT6 target gene (Supplementary Fig. 5). BCL6 expression was already barely detectable in untreated K562 cells, but expression was further reduced upon knockdown of LEF1 (Fig. 5G) and upon over expression of LEF1 (Fig. 5H). These data support the notion that LEF1 acts as an activator of *CCND1* expression in K562 cells and validate results by others^54^.

### LEF1 and PRMT6 are interdependent on the *CCND1* promoter

**Fig. 6:**
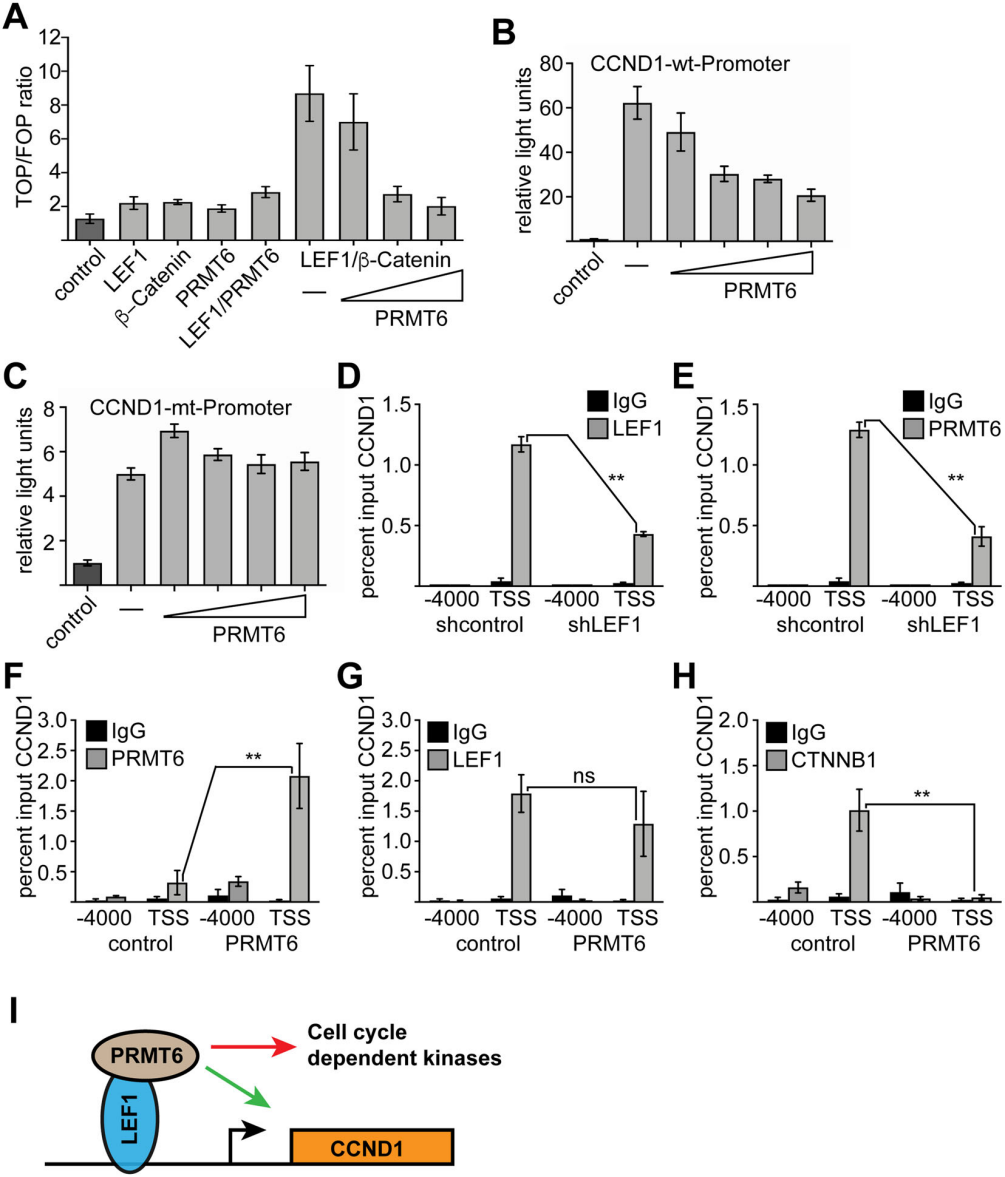
Interdependence of LEF1 and PRMT6 on *CCND1*. **A** PRMT6 TOP-Flash assay. The M50 Super 8x TOP-Flash^11^ was co-transfected with LEF1, PRMT6 and N89-(constantly active) β-Catenin^12^ into HEK293T cells. The negative control M51 Super 8× FOP-Flash was used for normalization. Co-transfection of LEF1 and N89-ß-Catenin leads to the highest TOP/FOP ratio. Increasing PRMT6 amount decreased the activating effect of β-Catenin**. B**, **C** *CCND1* luciferase assay. The bars represent the mean with standard deviation of two independent experiments, each measured in technical duplicates. **D**, **E** ChIP assay was performed seven days upon knockdown of LEF1 in K562 cells. Binding of LEF1 and PRMT6 to the *CCND1* promoter was reduced upon LEF1 knockdown. **F** ChIP assay upon over expression of PRMT6 in K562 cells. PRMT6 occupancy at the CCND1 promoter (transcription start site; TSS) was increased upon over expression of PRMT6. PCR with a primer localized at −4000 served as negative control. **G** ChIP assay with a LEF1 antibody upon over expression of PRMT6. LEF1 binding to the CCND1 promoter was not altered significantly. **H** ChIP assay revealed that binding of CTNNB1 (β-Catenin) was reduced upon over expression of PRMT6. Error bars represent the standard deviation from the mean. The *P*-values were calculated using Student’s *t*-test from at least three independent measurements. **P < 0.01. **I** Schematic representation of LEF1/PRMT6 activity on cell cycle genes.

LEF1 and PRMT6 are present on the *CCND1* promoter and influence CCND1 expression (Figs. 4 and Fig. 5). LEF1 is a transducer of wnt-signaling. In this context LEF1 acts as repressor of wnt target genes in the absence of β-catenin. To examine the potential connection between LEF1/β-catenin we performed a TOP/FOP reporter gene assay (Fig. 6A). Here the luciferase gene is driven by a promotor with six LEF1 binding sites. As control a variant is used in which these sites are mutated. In this assay LEF1, β-catenin, and PRMT6 slightly activated the reporter gene activity. Co-transfection of LEF1 with PRMT6 activated the reporter gene three-fold. Co-transfection of LEF1 with β-catenin led to eight-fold activation of the reporter gene. This activation was reduced with increasing amounts of co-transfected PRMT6. Subsequently, we analyzed the *CCND1* promoter in a reporter gene assay (Fig. 6B). The *CCND1* reporter construct displayed a sixty-fold activation compared to the empty reporter gene. Transfection of PRMT6 reduced the activity of the *CCND1* promoter in this context. A *CCND1* promoter construct with mutated LEF1 sites^55^ (Fig. 6C) (Supplementary Fig. 6), displayed reduced activity and was not influenced by co-transfection of PRMT6.

These data raised the question if LEF1 contributes to the recruitment of PRMT6 to the *CCND1* promoter. Thus, we performed a ChIP assay upon knockdown of LEF1. LEF1 binding to the *CCND1* promoter was reduced upon knockdown of LEF1 (Fig. 6D). This reduced LEF1 binding was associated with reduced presence of PRMT6 on the *CCND1* promoter (Fig. 6E). This supports the notion that LEF1 recruits PRMT6 to the promoter of *CCND1*.

The observation that PRMT6 expression reduced the activation of LEF1/β-catenin (Fig. 6A) raised the question if PRMT6 competes with β-catenin for LEF1 binding. To examine this notion, we over expressed PRMT6 in K562 cells. This increased PRMT6 occupancy at the CCND1 promoter (Fig. 6F), whereas LEF1 binding was not significantly altered (Fig. 6G). Interestingly, PRMT6 over expression led to a loss of β-catenin (CTNNB1) binding to the CCND1 promoter (Fig. 6H). Which indicates that PRMT6 and β-catenin compete for binding to the CCND1 promoter in this setting.

Taken together our data suggest that PRMT6 acts as an activator of *CCND1* expression in conjunction with LEF1 independently of β-catenin (Fig. 6I).

## Discussion

In the present study, we identified novel interaction partners of PRMT6 including the transcription factor LEF1. LEF1 recruits PRMT6 to cell cycle genes, in particular *CCND1* (Cyclin D1). Knockdown of PRMT6 and LEF1, respectively, reduced *CCND1* expression. Thus, the regulation of CCND1 by LEF1/PRMT6 is part of the growth regulating function of the proteins in hematopoietic cells.

PRMT6 lacks site specific DNA binding and is recruited to target loci by transcription factors. For the understanding of PRMT6 function it is critical to identify these interaction partners. Our approach identified several PRMT6 interactors, which are involved in distinct cellular processes. These include signaling molecules, RNA-binding proteins and transcription factors. Although, PRMT6 might be involved in global DNA-hypomethylation in cancer^11^ the transcriptional outcome of PRMT6-mediated H3R2 methylation is dependent on the genomic locus^10^. The later notion that PRMT6 acts locus dependent is supported by its specific activity on target genes in conjunction with transcription factors. This is exemplified by its association with PPARy in adipocyte differentiation^35^. Recently, we found that RUNX1 recruits PRMT6 to hematopoietic target genes and this way influences the balance between erythroid and megakaryocytic differentiation^8,25,36^. These data show that alterations in PRMT6 abundance does not only inhibit cell growth but also alters their differentiation. Our observation that LEF1 interacts with PRMT6 opens the possibility that PRMT6 is a general cofactor of LEF1 function, and might influence LEF1 target gene expression in distinct cell types.

In particular, we found that LEF1/PRMT6 target the *CCND1* gene, which encodes for LEF1 target gene cyclin D1^53^. Cyclin D1 is a major cell cycle regulator and a well-known oncogene in distinct tumor entities^52^. Accordingly, knockdown of PRMT6 led to down regulation of *CCND1* and an increase of cells in G1 phase of the cell cycle. Thus, our data well establish the notion that PRMT6 partly acts through *CCND1* on the cell cycle. Interestingly, knockdown of PRMT6 decreases *CCND1* expression. This has also been observed in U2OS cells upon knock down of PRMT6. In this study, knock down of PRMT6 resulted in increased binding of the ING2 repressive complex to the CCND1 locus^7^. However, in the same cell line no effect of PRMT6 knock down on CCND1 expression was detected^23^. Reduced CCND1 expression was also observed in PRMT6 −/− MEF cells^22^.

The knockdown of PRMT6 or LEF1 both led to decreased *CCND1* expression. Furthermore, ChIP experiments upon knockdown of LEF1 showed that also PRMT6 binding was lost. Thus, PRMT6 may act as a coactivator of LEF1. Because we did not observe coactivation by PRMT6 in transient reporter gene assays (Fig. 6), it is likely that its function requires native chromatin. PRMT6 is mostly described as an epigenetic repressor, which acts through H3R2me2a and this way negatively influences the establishment of the prominent H3K4me3 mark. However, PRMT6 can also activate gene expression^12,14^. These activities may be mediated through alternative sites of histone modifications, or by a modulating effect on interacting transcription factor. Although our data are in accordance with a positive role of a LEF1/PRMT6 complex on *CCND1* gene expression, one has to keep in mind that LEF1 activity is dependent on cellular signaling. Interesting in this respect, is our observation that PRMT6 interacts with the HMG-domain of LEF1, which mediates DNA-binding^49^, but not with the main binding site for β-catenin (Fig. 3H). This opens the possibility that β-catenin and PRMT6 could simultaneously interact with LEF1. However, our reporter gene assay shows that PRMT6 represses LEF1/β-catenin mediated activation (Fig. 6A). Furthermore, over expression of PRMT6 results in loss of CTNNB1 (β-catenin) enrichment at the CCND1 promoter (Fig. 6F–H). The later result implicates that CTNNB1 and PRMT6 compete for binding to LEF1. Because PRMT6 has distinct activities depending on the promoter context (Fig. 4), detailed analysis of LEF1/PRMT6 activity on different genes is of interest. Furthermore, to get more insight in role of PRMT6 in conjunction with wnt-signaling it would be interesting to study the involvement of PRMT6 on LEF1/β-catenin complex^39^ upon signaling.

Knockdown of PRMT6 reduced proliferation of K562 cells. This observation is in line with data showing that PRMT6 ablation decreases proliferation in the context of PRMT6 negative mouse embryonic fibroblasts^22^, and also in MCF7 and U2OS cells^21,23^. Interestingly, increased expression of PRMT6 is associated with a number of cancers. These observations open the possibility to treat cancer by targeting PRMT6. We have previously shown that knockdown of PRMT6 results in decreased proliferation of primary human CD34^+^ progenitor cells. As knockdown of PRMT6 in these cells led to a decreased number of colonies in a CFU assay^25^. Thus, an effect of PRMT6 inhibition on normal cells would be expected. However, the observation that PRMT6 knockdown does not lead to immediate cell death, but to G1 arrest or differentiation^25,35^, opens the possibility that there is a therapeutic window for PRMT6 inhibitors.

Taken together, we identified PRMT6 as a cofactor of the transcription factor LEF1. LEF1/PRMT6 regulate the expression of Cyclin D1. Thus, PRMT6 acts as a repressor of cell cycle dependent kinases^20,21,23^ and as an activator of Cyclin D1 (Fig. 6F). The further analysis of the interdependent network of PRMT6 associated transcription factors will further our understanding of proliferation control of normal and cancer cells. This will enable the rational development and usage of specific PRMT6 inhibitors for cancer therapy.

## Methods

### Cell culture

HEK293T/17 cells (ATCC no. CRL-11268) were cultured in DMEM GlutaMAX medium (GIBCO), K562 (ATCC no. CCL-243), HEL (ATCC no. TIB-180), TF-1a (ATCC no. CRL-2451), Jurkat (ATCC no. TIB-152), and U937 (ATCC CRL-1593.2) cells in RPMI 1640 GlutaMAX medium (GIBCO). Cells were tested as mycoplasma free. The cells were supplemented with 10% fetal calf serum (FCS) and 1% Penicillin/Streptomycin (GIBCO) and cultured at 37 °C in a 5% CO_2_ atmosphere.

### Xenograft mouse model for tumor growth

K562 cells were transduced with either a lentiviral control vector (shLacZ) or a lentiviral vector for knockdown of PRMT6 (shPRMT6#1 or shPRMT6#2). The lentiviral vectors were co-expressing GFP, as a detection control for the expression of the shRNA. After the transduction process, cells were sorted through fluorescence activated cell sorting (FACS) and expanded for 5 days. Subsequently cells were washed with PBS and 1 × 10^7^ cells were resuspended in a 1:1 matrigel/PBS-solution (100 µl cells in PBS + 100 µl Matrigel) and injected subcutaneously in the flank of 8 to 12-week-old mice (C57BL/6). The cohort of each group was seven mice, sample size was chosen based on preliminary data. No randomization or investigator blinding to the groups was performed. Data were analysed from all mice were included. The mice were observed for a time period of 24 days and tumor development was checked. Tumor size was measured 2–3 times a week. At the endpoint of the experiment mice were sacrificed through cervical dislocation, and the formed tumors were removed and microscopically investigated for GFP expression. Mice were maintained in the animal facility at the Georg–Speyer–Haus. Experiments were performed in accordance with German animal welfare legislation and were approved by the relevant authorities (Regierungspräsidium Darmstadt).

### Generation and production of lentiviral vectors

For lentiviral production, 1.4 × 10^7^ HEK293T/17 cells were seeded in T175 cell culture flasks and transfected after 24 hours with 144 µL PEI (1 mg/mL), 10 µg pMD2.G packaging plasmid, 18 µg pCMVdelta8.91 packaging plasmid and 25 µg of the transfer plasmid. Forty-eight hours after transfection, the supernatant was centrifuged at 400 × *g* for 5 min at 4 °C to remove cell debris and subsequently sterile filtered (PVDF, 0.45 µM). The supernatant was underlaid with 5 mL 20% sucrose and centrifugated at 20,000 rpm for 2.5 h at 4 °C. The pellet was resuspended in RPMI 1640 media. Over expression of LEF1 was performed with LeGO-iG2 vectors. Cloning was performed with standard methods. The reference sequences NP_057353.1 represents the full length LEF1 cDNA. Knockdown of *LEF1* and PRMT6 was performed with the help of pGIPZ vectors. ShRNA sequences are given (Supplementary Table 3).

### Transduction

For transduction of cell lines 1 × 10^5^ K562 cells were seeded in 24-well plates with 250 µL *RPMI 1640* medium and incubated for 4 h. Subsequently, 200 µL virus and protaminsulfate (6 µg/mL) was added and spinoculation was performed at 1200 × *g* for 60 min at 32 °C.

### Avi-streptavidin purification

Transduced cells expressing the avi-PRMT6 protein were grown in heavy (H) SILAC medium and the control cells with avi-tag only were grown in light (L) SILAC medium. Nuclear extracts of 1 × 10^8^ K562 avi-PRMT6 and Bio-tag only control cells were prepared as described^56^. Streptavidin Beads (Dynabeads M-280, Life Technologies) were used for protein pull down of avi-tagged PRMT6 protein and avi-tag only control. The beads were washed five times (10 mM Tris (pH 7.5); 0.2 M NaCl; 10% Glycerol; 0.5 mM DTT; 0.1% NP-40. The proteins were eluted from the beads with 27 µL 4× NuPAGE LDS Sample Buffer and 3 µL 4× NuPAGE Reducing Agent at 95 °C for 5 min. Avi-PRMT6 and avi-tag only control samples were combined and suspected to mass spectrometry.

### Mass spectrometry

Mass spectrometry raw data were analyzed with the *MaxQant* software (version 1.5.2.8). Proteins with a normalized ratio *H*/*L* of >2 were defined as possible members of the PRMT6 interactome. Details are given in Supplementary Material and Methods. The list of identified proteins is shown in Supplementary Table 1. The mass spectrometry proteomics data have been deposited to the ProteomeXchange Consortium via the PRIDE^57^ partner repository with the dataset identifier PXD021804.

### Co-Streptavidin precipitation

For Co-Streptavidin precipitation (CoSP), 2 × 10^6^ HEK293T cells per well were seeded in 6-well plates. Cells were transfected with 2 µg plasmid and 7.5 µL PEI per well. 48 hours after transfection, the cells were resuspended in 500 µL lysis buffer (50 mM Tris pH 7.4, 50 mM NaCl, 1% v/v Triton X-100), incubated for 30 min on ice and centrifuged at 13,000 rpm for 10 min at 4 °C. Twenty-five microliter of lysate were removed and used as input sample. Biotin tagged PRMT6 protein was pulled out with Streptavidin Beads (Dynabeads M-280, Life Technologies) and washed six times with lysis buffer. The proteins were eluted from the beads with 20 µL undiluted NuPAGE LDS Sample Buffer at 95 °C for 5 min and analysed with Western Blot.

### GST pulldown assay

For GST-pulldown, GST-fusion proteins were constructed using pGEX-4T1 (Amersham Biosciences). GST or GST-fusion proteins were co-expressed with chaperone plasmid pGro7 (TAKARA) in *E. coli* BL21 (DE3) (NEB). Induction was performed by using 0.05% l-Arabinose (pGro7) and 1 mM IPTG (pGEX-4T1) for 4 h at 37 °C. Cells were harvested in lysis buffer (50 mM Tris, pH 7.4, 100 mM NaCl, 10% Glycerol, 0.1% Triton X-100, 1 mM DTT, 1 mM EDTA, protease inhibitor), disrupted by sonification and the supernatant was incubated with glutathione beads (PierceTM, Thermo Scientific) for 4 h with rotation at 4 °C. Glutathione beads were washed for three times with lysis buffer and then incubated in dissociation buffer (50 mM Tris, pH 7.4, 100 mM KCl, 10 mM MgCl_2_, 5 mM ATP) for 2 h at 4 °C to remove the non-specific binding of co-expressed chaperone proteins. The Beads were washed two times with lysis buffer and equal amounts of protein bound to beads were employed in GST-pulldown assay. GST-pulldown with GST-PRMT6 as bait was either performed with LEF1 protein expressed in HEK293T cells or with in vitro translated LEF1. For expression of LEF1 protein in HEK293T cells, 2 × 10^6^ cells were seeded into a 6-cm dish. Cells were transfected with 6 μg Plasmid and 18 μL of Metafectene transfection reagent. After 48 h, cells were harvested and lysed. For each pulldown reaction 500 μg of cell lysate was used. In vitro translation was performed using the TNT T7 Quick coupled transcription/translation system (Promega). For pulldown reaction, 10 μL of in vitro translate was incubated with protein beads in 250 μL lysis buffer for 3 h at 4 °C. With the same buffer, protein beads were washed for four times and boiled in 20 μL of SDS loading dye. The eluted proteins were analyzed with Western Blot. For radioactive labeling of protein, in vitro transcription/translation was performed in the presence of ^35^S-methionine (10 mCi/ml; 1000 Ci/mmol; Hartmann Analytic). Proteins were pulled out with glutathione beads (see above), detected by SDS-PAGE and autoradiography as described^58^.

### Gene expression analysis

Total RNA was isolated of 2 × 10^6^ cells using the RNeasy Mini Kit (Quiagen, Hilden, Germany). Complementary DNA was generated with the PrimeScript RT Master Mix Kit (Takara Bio Europe AB). Gene expression analysis was performed with SYBR Green PCR Mastermix (Eurogentec, Luettich, Belgium) on a LightCycler 480 (Roche, Mannheim, Germany). Glyceraldehyde-3-phosphate dehydrogenase (GAPDH) expression was used for normalization. DNA oligonucleotides used for the qPCR analysis are listed (Supplementary Table 4). The error bars represent the standard deviation from the mean of three independent determinations. Only experiments which could be reproduced in biological duplicates were included. Knockdown of PRMT6 in K562 cells and subsequent genome wide expression analysis has been described^25^.

### Chromatin immunoprecipitation (ChIP)

Cell culture cross-linking and chromatin digestion was performed according to the SimpleChIP protocol from *Cell Signaling Technology*. Chromatin immunoprecipitation assays were performed according to the *X-ChIP* protocol from *Abcam*. The specific antibodies and the concentration are listed (Supplementary Table 5). The DNA was concentrated with the *ChIP DNA Clean & Concentrator Kit* (*Zymo Research*) and analysed by ChIP qPCR. DNA oligonucleotides used for the ChIP PCR analysis are listed (Supplementary Table 4). Only experiments were included, which could be reproduced with two different chromatin preparations.

### ChIP sequencing analysis

LEF1 ChIP-seq data from human K562 cells (GEO accession number GSE105908) was retrieved from the ENCODE Project^50,51^. The LEF1 peak coordinates from file ENCFF043YZF were reduced to a three columns BED file format. The Bioconductor package ChIPpeakAnno version 3.10^59^ was used for peak annotation and data analysis. Code availability: The R script is provided in Supplementary Fig. 7.

### Luciferase Assay

For luciferase assays, 9 × 10^4^ HEK293T cells per well were seeded in 24-well plates. Cells were transfected with 1000 ng plasmid and 2.5 µL PEI per well. Forty-eight hours after transfection, the cells were resuspended in 90 µL lysis buffer, incubated for 20 min on ice and centrifuged at 13,000 rpm for 10 min at 4 °C. Ten microliter lysate was mixed with 100 µL luciferase buffer (21.625 mM Glycyl-Glycine, 1 mM ATP, 0.075 mM Luciferin, 10 mM MgSO_4_) and a Victor X4 Multiple Plate Reader (PerkinElmer) was used for bioluminescence measurement. Transfection efficiency was normalized with co-transfected β-galactosidase. Ten microliter lysate was mixed with 100 μL of buffer (11.1 mM MgCl2, 50 mM β-Mercaptoethanol, 3.25 mM o-Nitrophenyl-β-d-galactopyranosid, 74.4 mM sodium phosphate). Absorption was measured after 5 min at 420 nm. The error bars represent the standard deviation from the mean of three independent determinations.

### Western blotting

Protein samples were analysed in SDS-PAGE and transferred with a semi-dry system (Biorad) using standard techniques. Primary antibodies and dilutions are listed in Supplementary Table 5. The Blots were analysed with the Odyssey® CLx Imaging System (LI COR Biosciences). IRDye800CW secondary antibodies (LI COR Biosciences) were used in a dilution of 1:15,000. Full Western Blots are shown in Supplementary Material.

### Cell cycle analysis

For cell cycle analysis, 500,000 cells were washed with 500 µL PBS and centrifuged at 400 × *g* for 5 min at RT. The cells were resuspended in 500 μL ice cold 70% ethanol and fixed for 30 min on ice. The fixed cells were centrifuged at 1200 × *g* for 5 min at 4 °C and washed twice in 500 μL PBS. The cells were resuspended in 500 µL DAPI staining solution (PBS + 0.1% Triton X + 10 µg/mL DAPI) and incubated for 30 min at RT. Cells were resuspended in 500 µL PBS.

### Statistics

Sample size was determined based on previous publications and the variability observed in preliminary experiments. Experiments were performed in at least three in dependent replicates and were analysed using the *GraphPad Prism* software. Data are presented as mean ± standard error. Statistical significance was calculated with Student’s *t*-test or ANOVA (analysis of variance), variance was equal between compared groups. *P*-values are noted as: *P < 0.05; **P < 0.01; ***P < 0.001. A *P*-value < 0.05 was considered statistically significant.

## Supplementary information

Supplementary Tables

Supplemental Figures

## Data Availability

Knockdown of PRMT6 in K562 cells and subsequent genome wide expression analysis has been described^25^. Data were deposited to the GEO-databank (Accession: GSE92251). The mass spectrometry proteomics data have been deposited to the ProteomeXchange Consortium via the PRIDE^57^ partner repository with the dataset identifier PXD021804.
