# Supplementary material for: PRMT6 activates cyclin D1 expression in conjunction with the transcription factor LEF1
Source: Oncogenesis. 2021 May 17;10(5):42. doi: 10.1038/s41389-021-00332-z (PMC8129428; doi:10.1038/s41389-021-00332-z)
Supplement: Supplementary file 2 — Supplemental Figures [file 41389_2021_332_MOESM2_ESM.pdf]

Supplementary Figure 1

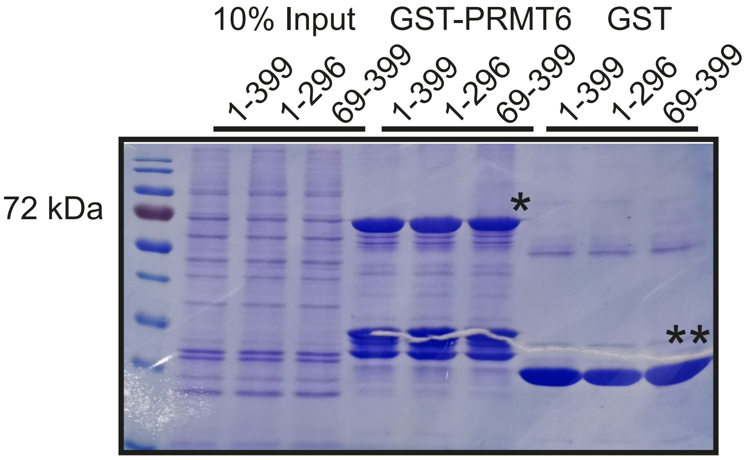

Supplementary Figure 1. Coomassie staining of the gel from the GST-pulldown (Figure 3H).

* GST-PRMT6

** GST

Supplementary Figure 2

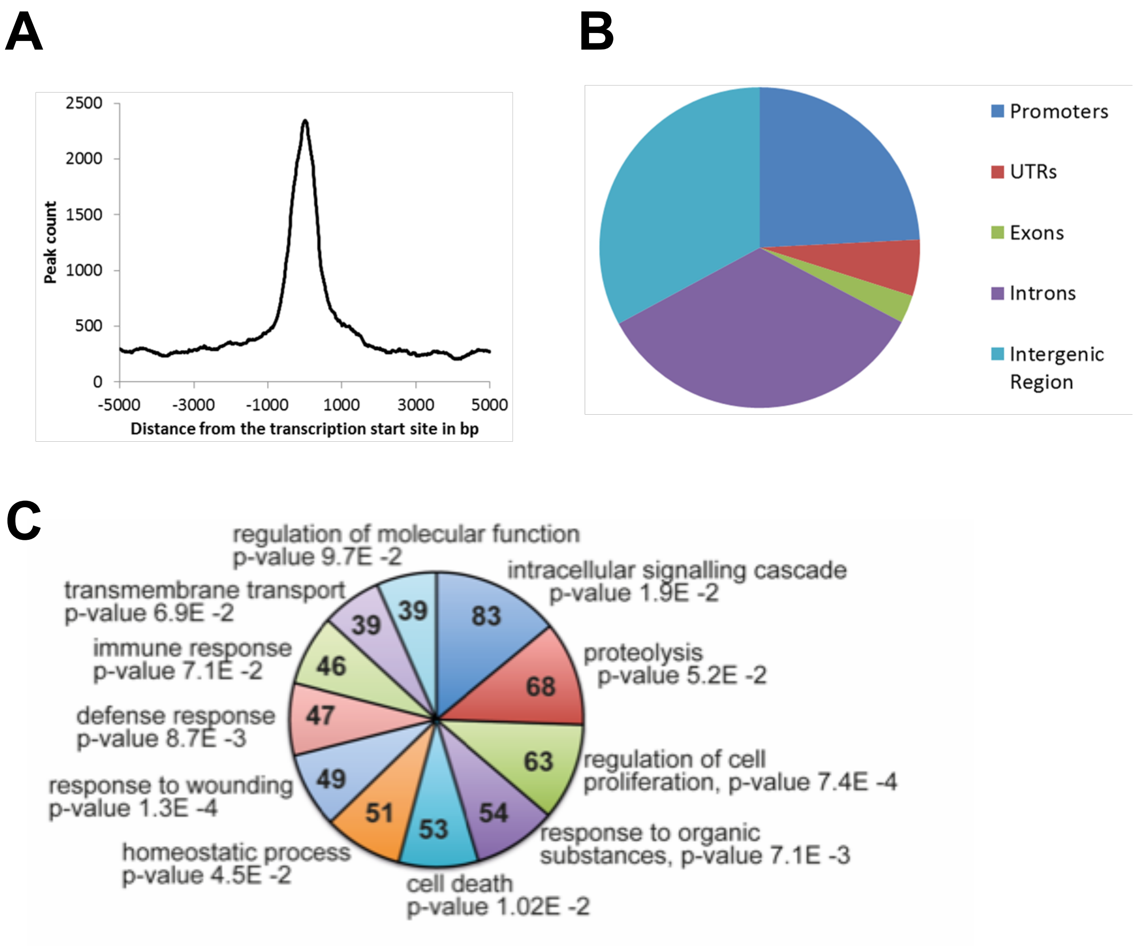

Supplementary Figure 2. LEF1 ChIP-Seq analysis in K562 cells. **A** Distribution of the aggregated peak numbers around the transcription start site. The distance from the TSS is shown in bp. The peak count shows that most LEF1 peaks are located near a transcription start site. **B** Peak distribution over different genomic features. 24.2% of the peaks are located in promoter regions or introns. 32.9% are located in intergenic regions and 42.9% in UTRs, Exons and Introns.

C GO-term analysis of genes with altered expression upon PRMT6 knock down in K562 cells. PRMT6 was knocked down by shRNA and gene expression changes were measured by Agilent whole human genome expression microarrays. Altered genes were grouped according to their GO-terms using DAVID. Enriched GO-terms and the number of genes in the groups are shown.

Supplementary Figure 3

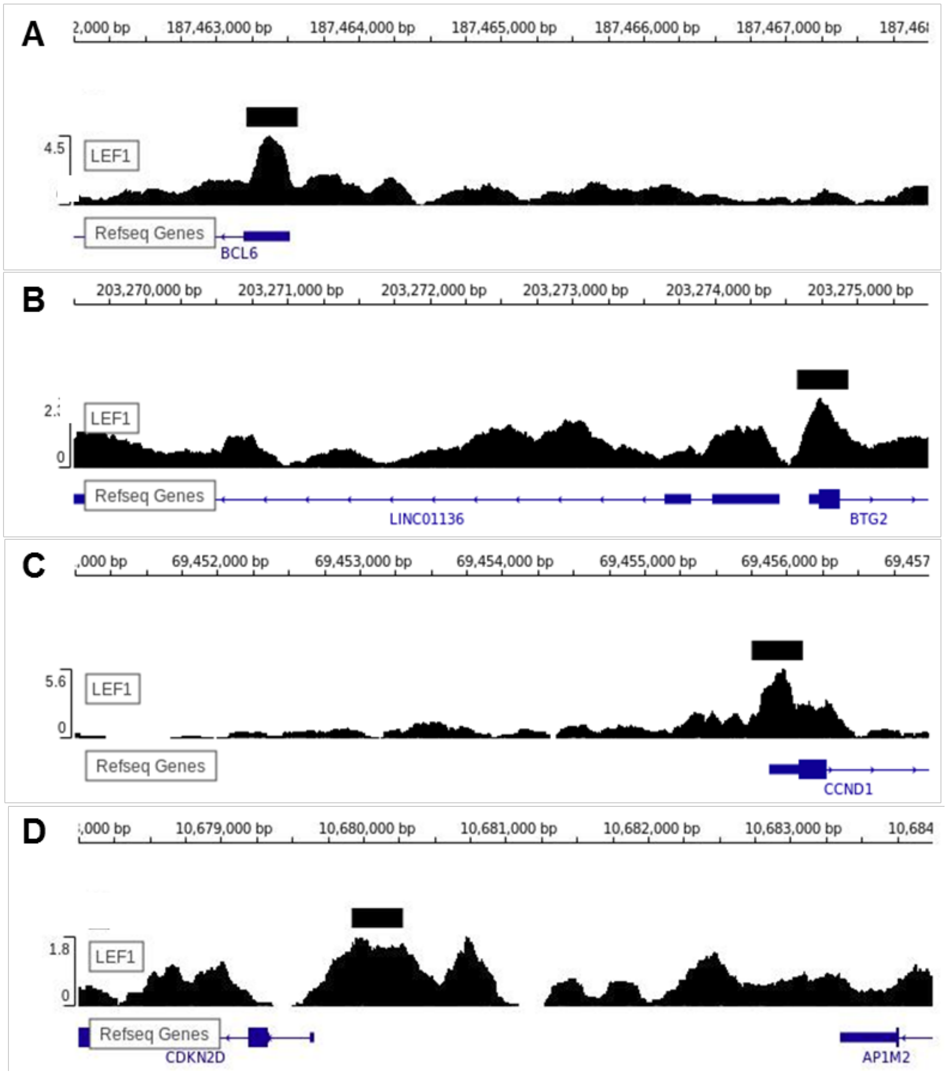

Supplementary Figure 3. LEF1 peaks in K562 cells. **A.** TSS of BCL6 (chr3:187,462,001-187,468,000) **B.** TSS of BTG2 (chr1:203,269,501-203,275,500) **C.** TSS of CCND1 (chr11:69,451,001-69,457,000) **D.** TSS of CDKN2D (chr19:10,678,001-10,684,000)

Supplementary Figure 4

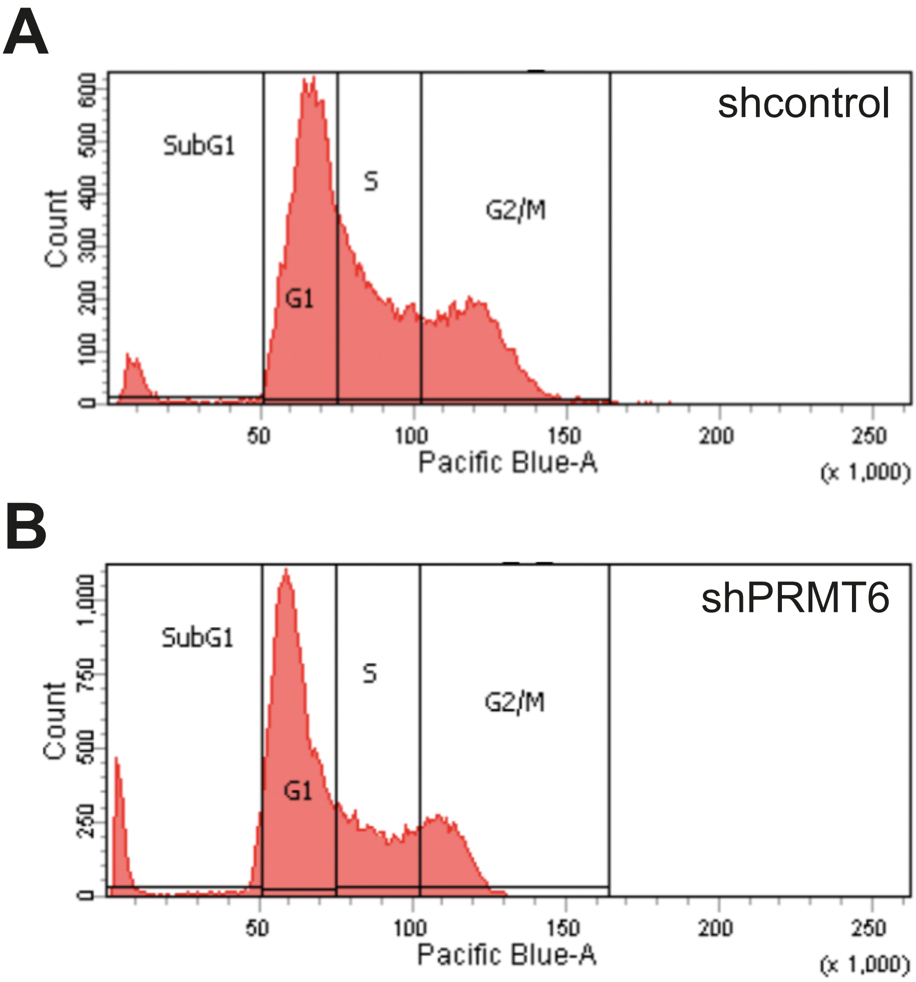

Supplementary Figure 4. Cell cycle analysis of transduced K562 cells. The cells were fixed with 70 % ethanol, the double stranded DNA was DAPI stained. **A.** Normal cell cycle progression of K562 cells transduced with the empty vector control**. B.** Aberrant cell cycle progression of K562 cells transduced with shPRMT6. G1 (Gap 1); (Synthesis) S; G2 (Gap 2); M (mitosis).

Supplementary Figure 5

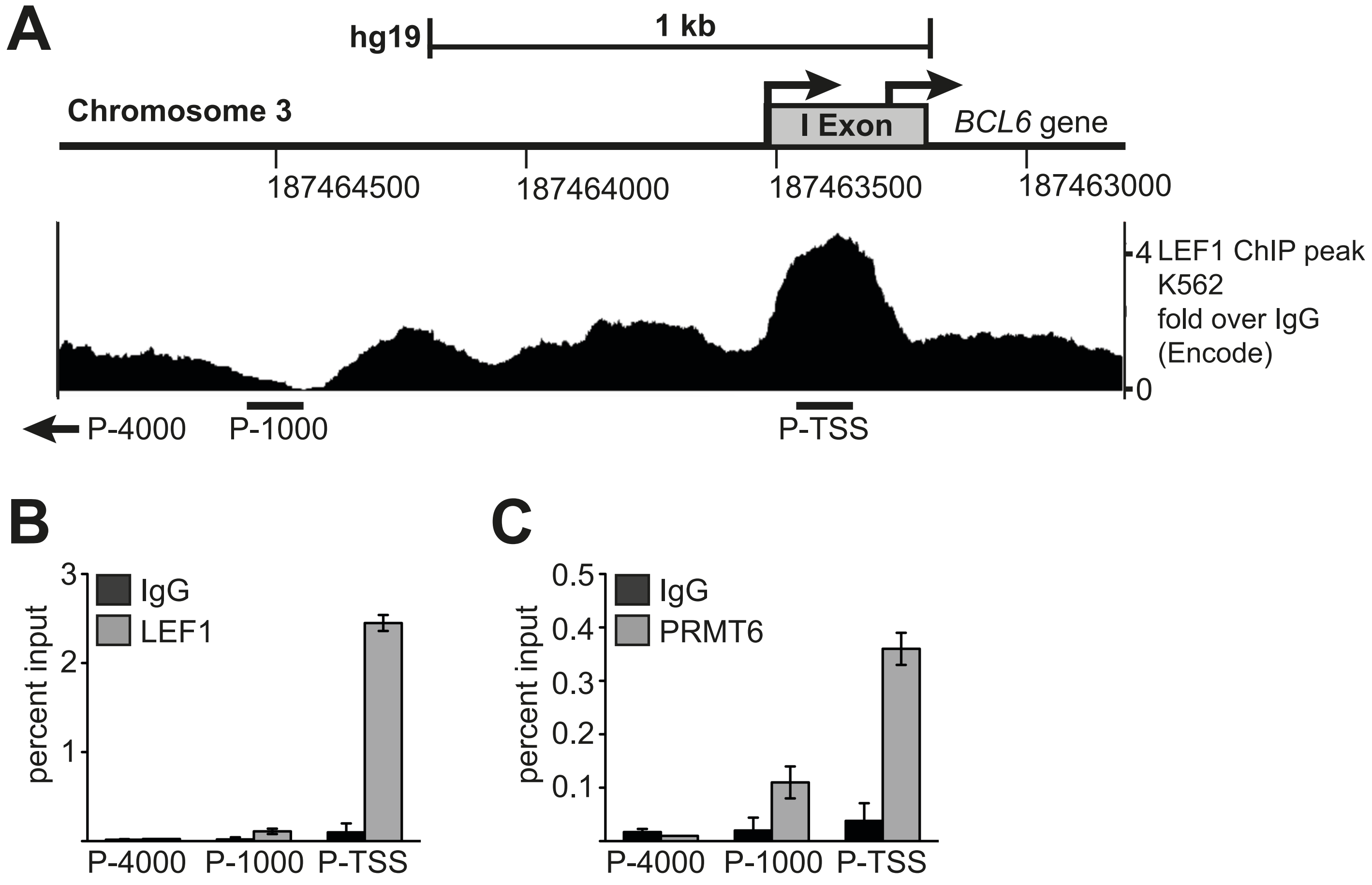

Supplementary Figure 5. A. Schematic of the BCL6 locus. The LEF1 peak in K562 cells is shown. Positions of ChIP Primer are shown. **B.** LEF1 ChIP shows LEF1 binding at the TSS of *BCL6.* **C.** PRMT6 ChIP shows PRMT6 binding close to the TSS of *BCL6.*

Supplementary Figure 6

```
                    10        20        30        40        50        60        70        80        90       100
           ....|....|....|....|....|....|....|....|....|....|....|....|....|....|....|....|....|....|....|....|
CCND1 WT   GATATGGGGTGTCGCCGCGCCCCAGTCACCCCTTCTCGTGGTCTCCCCAGGCTGCGTGTGGCCTGCCGGCCTTCCTAGTTGTCCCCTACTGCAGAGCCAC
CCND1 mut  GATATGGGGTGTCGCCGCGCCCCAGTCACCCCTTCTCGTGGTCTCCCCAGGCTGCGTGTGGCCTGCCGGCCTTCCTAGTTGTCCCCTACTGCAGAGCCAC

                   110       120       130       140       150       160       170       180       190       200
           ....|....|....|....|....|....|....|....|....|....|....|....|....|....|....|....|....|....|....|
CCND1 WT   CTCCACCTCACCCCCTAAATCCCGGGGGACCCACTCGAGGCGGACGGGGCCCCCTGCACCCCTCTTCCCTGGCGGGGAGAAAGGCTGCAGCGGGGCGATT
CCND1 mut  CTCCACCTCACCCCCTAAATCCCGGGGGACCCACTCGAGGCGGACGGGGCCCCCTGCACCCCTCTTCCCTGGCGGGGAGAAAGGCTGCAGCGGGGCGATT

                   210       220       230       240       250       260       270       280       290       300
           ....|....|....|....|....|....|....|....|....|....|....|....|....|....|....|....|....|....|....|
CCND1 WT   TGCATTTCTATGAAAACCGGACTACAGGGGCAACTCCGCCGCAGGGCAGGCGCGGCGCCTCAGGGATGGCTTTTGGGCTCTGCCCCTCGCTGCTCCCGGC
CCND1 mut  TGCATTTCTATGAAAACCGGACTACAGGGGCAACTCCGCCGCAGGGCAGGCGCGGCGCCTCAGGGATGGCTTTTGGGCTCTGCCCCTCGCTGCTCCCGGC

                   310       320       330       340       350       360       370       380       390       400
           ....|....|....|....|....|....|....|....|....|....|....|....|....|....|....|....|....|....|....|
CCND1 WT   GTTTGGCGCCCGCGCCCCCTCCCCCTGCGCCCGCCCCCGCCCCCCTCCCGCTCCCATTCTCTGCCGGGCTTTGATCTTTGCTTAACAACAGTAACGTCAC
CCND1 mut  GTTTGGCGCCCGCGCCCCCTCCCCCTGCGCCCGCCCCCGCCCCCCTCCCGCTCCCATTCTCTGCC--G----G------G--TAACAACAGTAACGTCAC

                   410       420       430       440       450       460       470       480       490       500
           ....|....|....|....|....|....|....|....|....|....|....|....|....|....|....|....|....|....|....|
CCND1 WT   ACGGACTACAGGGGAGTTTTGTTGAAGTTGCAAAGTCCTGGAGCCTCCAGAGGGCTGTCGGCGCAGTAGCAGCGAGCAGCAGAGTCCGCACGCTCCGGCG
CCND1 mut  ACGGACTACAGGGGAGTTTTGTTGAAGTTGCAAAGTCCTGGAGCCTCCAGAGGGCTGTCGGCGCAGTAGCAGCGAGCAGCAGAGTCCGCACGCTCCGGCG

                   510       520       530       540       550       560       570       580
           ....|....|....|....|....|....|....|....|....|....|....|....|....|....|....|....|....
CCND1 WT   AGGGGCAGAAGAGCGCGAGGGAGCGCGGGGCAGCAGAAGCGAGAGCCGAGCGCGGACCCAGCCAGGACCCACAGCCCTCCCCAG
CCND1 mut  AGGGGCAGAAGAGCGCGAGGGAGCGCGGGGCAGCAGAAGCGAGAGCCGAGCGCGGACCCAGCCAGGACCCACAGCCCTCCCCAG
```

Supplementary Figure 6. Sequence of the CCND1 Promoter. CCND1 WT: -962 human cyclin D1 promoter pGL3Basic (Addgene Plasmid #32727). CCND1 mut: -962 human cyclin D1 promoter TCF (1)(2) sites mutant pGL3Basic (Addgene Plasmid #32734). The two adjacent LEF1 consensus sequences are removed in the mutated CCND1 promoter. Tetsu, McCormick 1999

Supplementary Figure 7

```r
#Install the following libraries
library(ChIPpeakAnno)
library(EnsDb.Hsapiens.v75)
library(TxDb.Hsapiens.UCSC.hg19.knownGene)
library(org.Hs.eg.db)

#Transfer the ChIP-peaks to GRanges
annoData <- toGRanges(EnsDb.Hsapiens.v75, feature="gene")
macs <- system.file("extdata", "K562_conservative_diss.txt", package="ChIPpeakAnno")
K562_peaks <- toGRanges(macs, format="BED")

#Extract the peaks overlapping the TSS +/- 500 bp
overlaps.anno <- annotatePeakInBatch(K562_peaks,
                                     AnnotationData=annoData,
                                     output="nearestBiDirectionalPromoters",
                                     bindingRegion=c(-500, 500))

#Add the Entrez ID to the peaks
overlaps.anno <- addGeneIDs(overlaps.anno,
                            "org.Hs.eg.db",
                            IDs2Add = "entrez_id")

write.csv(as.data.frame(unname(overlaps.anno)), "anno_K562_diss.csv")

#Calculate the enriched GO-terms
over <- getEnrichedGO(overlaps.anno, orgAnn="org.Hs.eg.db",
                      maxP=.05, minGOterm=10,
                      multiAdjMethod="BH", condense=TRUE)

write.csv(as.data.frame(unname(over[["bp"]][, -c(8, 16)])), "over_K562_diss.csv")

#Calculate the peak distribution
write.csv(as.data.frame(unname(binOverFeature(K562_peaks, annotationData=annoData,
                                              radius=5000, nbins=500, FUN=length,
                                      errFun=0, ylab="count")
            )),"K562_bins_diss.csv")

#Calculate the distribution over the Chromosome Regions
aCR<-assignChromosomeRegion(K562_peaks, nucleotideLevel=FALSE,
                            precedence=c("Promoters", "immediateDownstream",
                                         "fiveUTRs", "threeUTRs",
                                         "Exons", "Introns"),
                            TxDb=TxDb.Hsapiens.UCSC.hg19.knownGene)

write.csv(as.data.frame(unname(aCR)), "K562_Chr_region.csv")
```

Supplementary Figure 7: Command line arguments of the R script. The MACS peak coordinates were reduced to a 3 columns BED file and transferred to Granges with the Bioconductor package ChIPpeakAnno, (V.3.10). Only peaks which overlap +/- 500 bp around the transcription start site were extracted. The peaks were annotated with entrez IDs and the enriched GO-terms were calculated.

Supplementary Material and Methods

Glutathione S-transferase (GST) pulldown assay

For GST-pulldown, GST-fusion proteins were constructed using pGEX-4T1 (Amersham Biosciences). GST or GST-fusion proteins were co-expressed with chaperone plasmid pGro7 (TAKARA) in *E. coli* BL21 (DE3) (NEB). Induction was performed by using 0.05% L-Arabinose (pGro7) and 1 mM IPTG (pGEX-4T1) for 4 hours at 37°C. Cells were harvested in lysis buffer (50 mM Tris, pH 7.4, 100 mM NaCl, 10 % Glycerol, 0.1 % Triton X-100, 1 mM DTT, 1 mM EDTA, protease inhibitor), disrupted by sonification and the supernatant was incubated with glutathione beads (Pierce™, Thermo Scientific) for 4 hours with rotation at 4°C. Glutathione beads were washed for three times with lysis buffer and then incubated in dissociation buffer (50 mM Tris, pH 7.4, 100 mM KCl, 10 mM $MgCl_2$, 5 mM ATP) for 2 hours at 4°C to remove the non-specific binding of co-expressed chaperone proteins. The Beads were washed two times with lysis buffer and equal amounts of protein bound to beads were employed in GST-pulldown assay. GST-pulldown with GST-PRMT6 as bait was either performed with LEF1 protein expressed in HEK293T cells or with *in vitro* translated LEF1.

For expression of LEF1 protein in HEK293T cells, 2 x 10^6 cells were seeded into a 6-cm dish. Cells were transfected with 6 µg Plasmid and 18 µl of Metafectene transfection reagent. After 48 hours, cells were harvested and lysed. For each pulldown reaction 500 µg of cell lysate was used.

In vitro translation was performed using the TNT T7 Quick coupled transcription/translation system (Promega). For pulldown reaction, 10 µl of *in vitro* translate was incubated with protein beads in 250 µl lysis buffer for 3 hours at 4°C. With the same buffer, protein beads were washed for four times and boiled in 20 µl of SDS loading dye. The eluted proteins were analysed with Western Blot.

For radioactive labelling of protein, *in vitro* transcription/translation was performed in the presence of ^{35}S-methionine (10 mCi/ml; 1000 Ci/mmol; Hartmann Analytic). Proteins were pulled out with glutathione beads (see above), detected by SDS-PAGE and autoradiography as described [39].

Mass spectrometry

Transduced cells expressing the avi-PRMT6 protein were grown in heavy (H) SILAC medium and the control cells with avi-tag only were grown in light (L) SILAC medium. Nuclear extracts of 1 × 10^8 K562 avi-PRMT6 and Bio-tag only control cells were prepared. Streptavidin Beads (Dynabeads M-280, Life Technologies) were used for protein pull down of avi-tagged PRMT6 protein and avi-tag only control. The beads were washed five times (10 mM Tris (pH 7,5); 0.2 M NaCl; 10% Glycerol; 0.5 mM DTT; 0.1 % NP-40. The proteins were eluted from the beads with 27 µL 4x NuPAGE LDS Sample Buffer and 3 µL 4x NuPAGE Reducing Agent at 95 °C for 5 min. avi-PRMT6 and avi-tag only control samples were combined and the protein mixture was subjected to polyacrylamide gel electrophoresis. The separated proteins were stained with Coomassie Brilliant Blue and the whole gel lane was excised as 23 individual bands, which were subjected to in-gel digestion with trypsin.

The peptide mixtures were analyzed on a Q Exactive HF orbitrap mass spectrometer (Thermo Fisher Scientifc) coupled to an Ultimate 3000 RSLCnano HPLC system (Thermo Fisher Scientific). The peptides were first trapped on a precolumn (ReproSil-Pur 120 C18-AQ, 5 µm; Dr. Maisch GmbH; 100 µm x 5 cm; self-packed) and then separated on an analytical column (ReproSil-Pur 120 C18-AQ, 3 µm; Dr. Maisch GmbH; 350 x 0.075 mm; self-packed) with a 1 hour linear gradient of 2-40% solvent B [80% (vol/vol) ACN, 0.1% FA] and versus solvent A (0.1% FA in water) at a constant flow rate of 300 nL·min-1. Eluting peptides were analyzed by data-dependent acquisition using a top 30 MS/MS method with a survey scan resolution setting of 60,000 FWHM and an MS/MS resolution setting of 15,000 FWHM at 200 *m/z*. The 30 most abundant ions within the 350-1600 *m/z* range were selected for HCD with an NCE setting of 28% and an isolation width of 1.6 *m/z*. AGC target values and maximum ion injection times for MS and MS/MS were set to 10^6 in 50 ms and 10^5 in 60 ms, respectively. Selected precursor mass-to-charge ratio values were dynamically excluded from fragmentation for 30 s.

MS data were processed using the MaxQuant software (version 1.5.2.8, MPI for Biochemistry). Fragment ion mass spectra were searched against the UniProtKB/Swiss-Prot human protein database (date: November 2016) supplemented with 245 frequently observed contaminants using the integrated Andromeda search engine. The mass tolerances for precursor and fragment ions were set to 6 and 20

ppm after initial recalibration, respectively. Oxidation of methionine and acetylation on the protein *N*-terminus were considered as variable modifications. Carbamidomethylation of cysteine was defined as a fixed modification. Minimal peptide length was set to seven amino acids, allowing up to two missed cleavages. Both on the peptide and protein level the false discovery rate (FDR) was set to 1% on using a forward-and-reverse concatenated decoy database approach. SILAC multiplicity was set to double labeling (Lys+0/Arg+0, Lys+8/Arg+10) requiring at least two ratio counts for peptide quantitation and enabling the “re-quantify” option.

Related to Figure 1

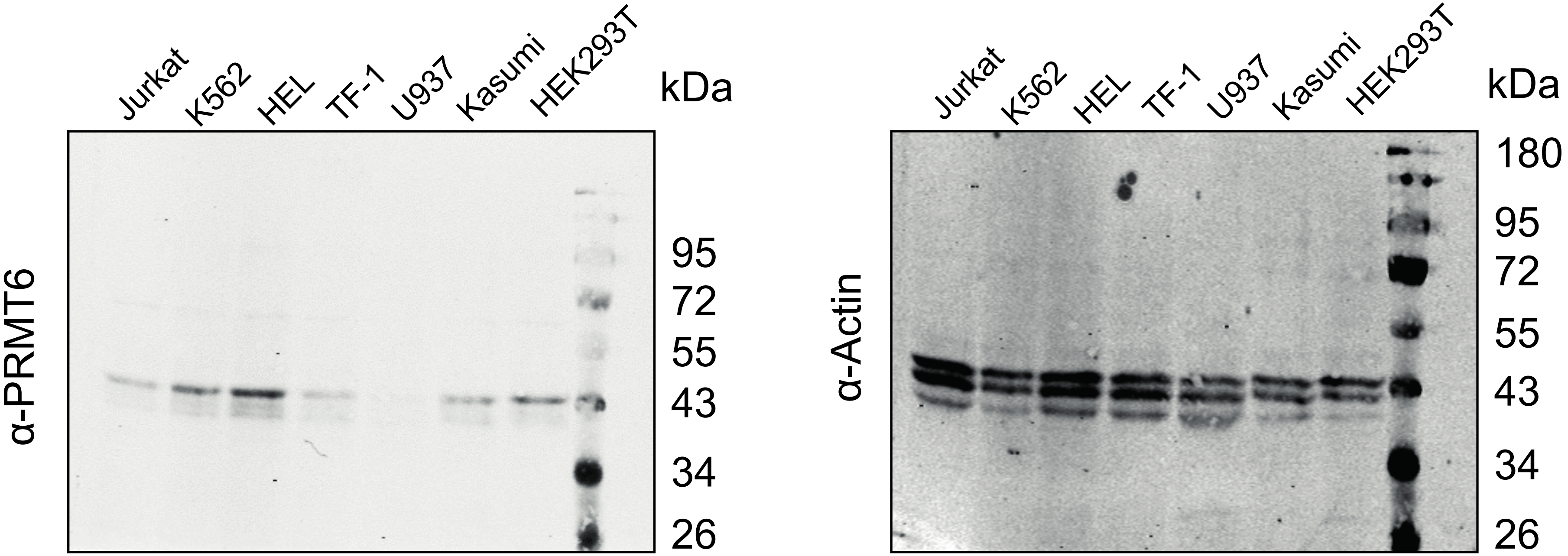

Related to Figure 2A

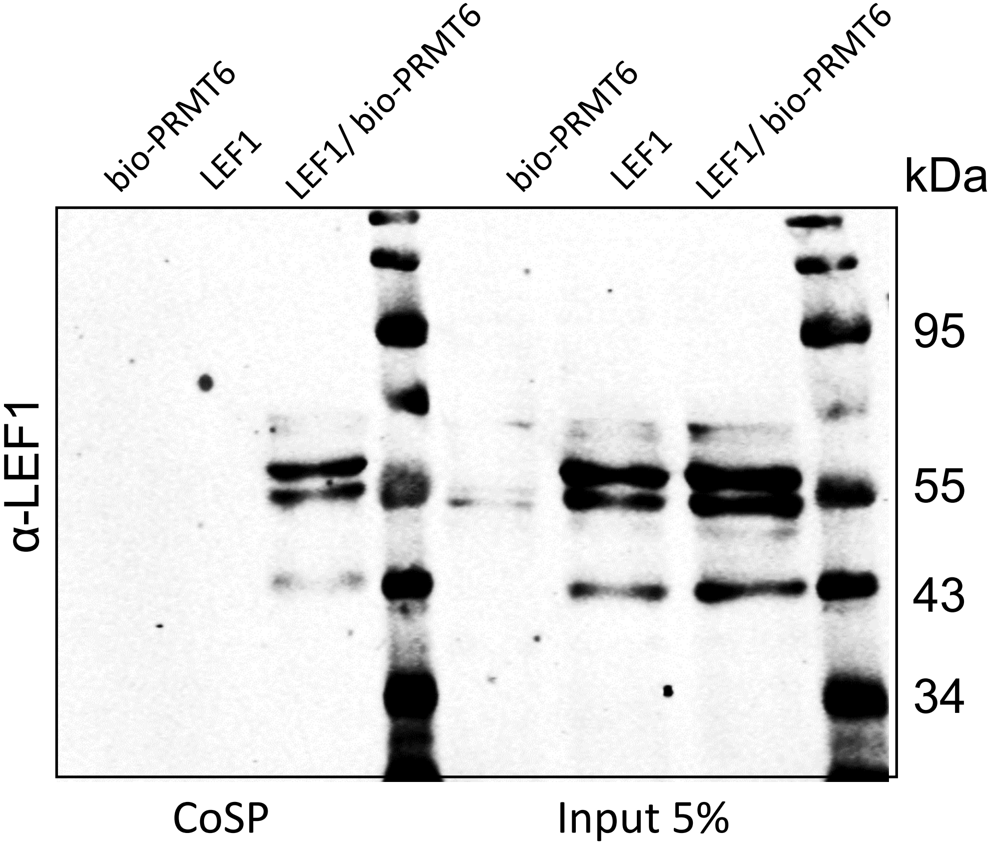

Related to Figure 2A

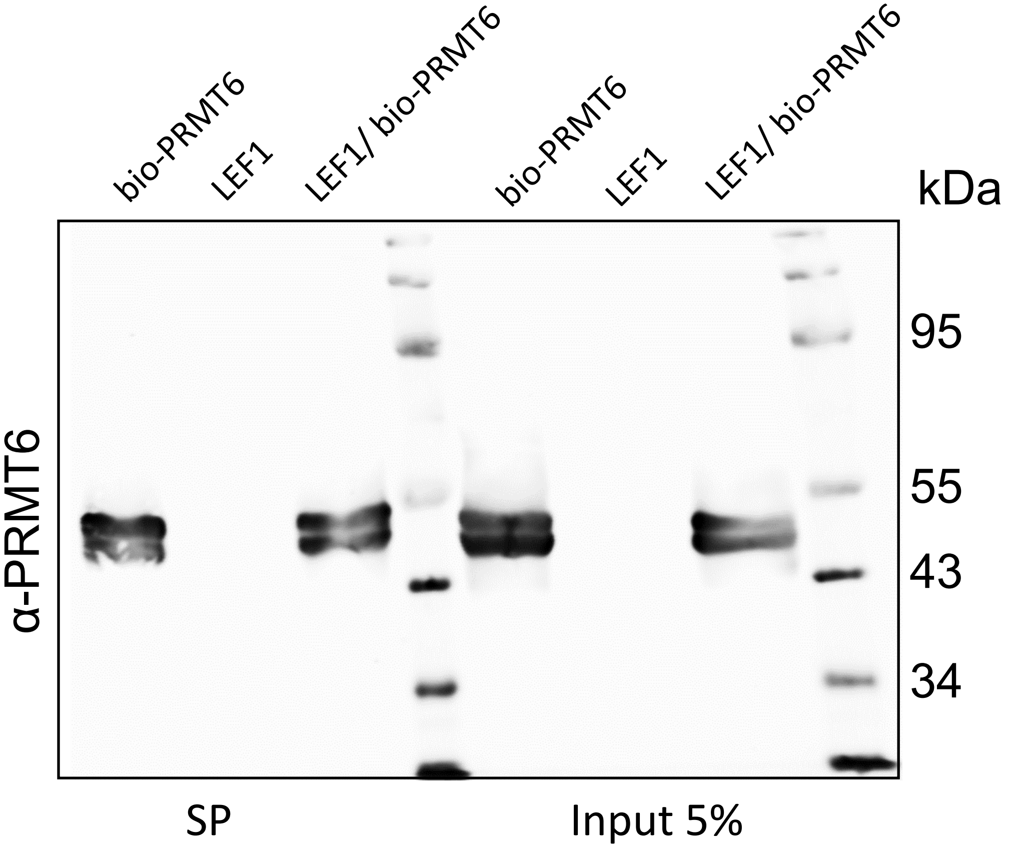

Related to Figure 2B

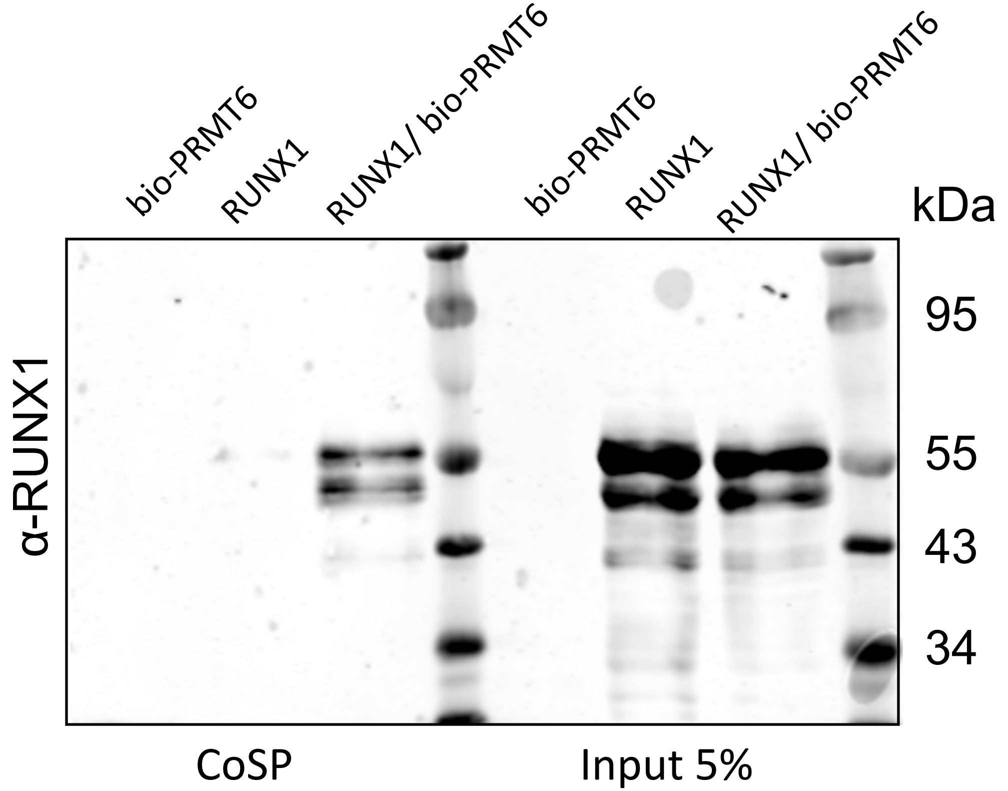

Related to Figure 2B

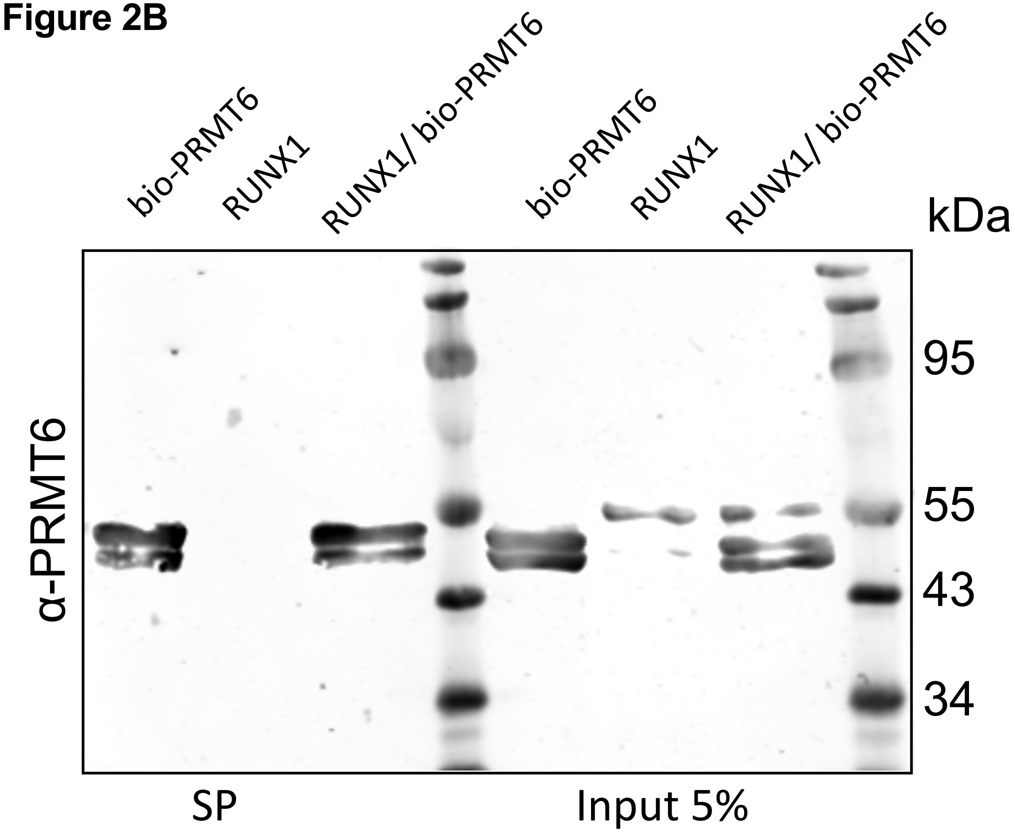

Related to Figure 2C

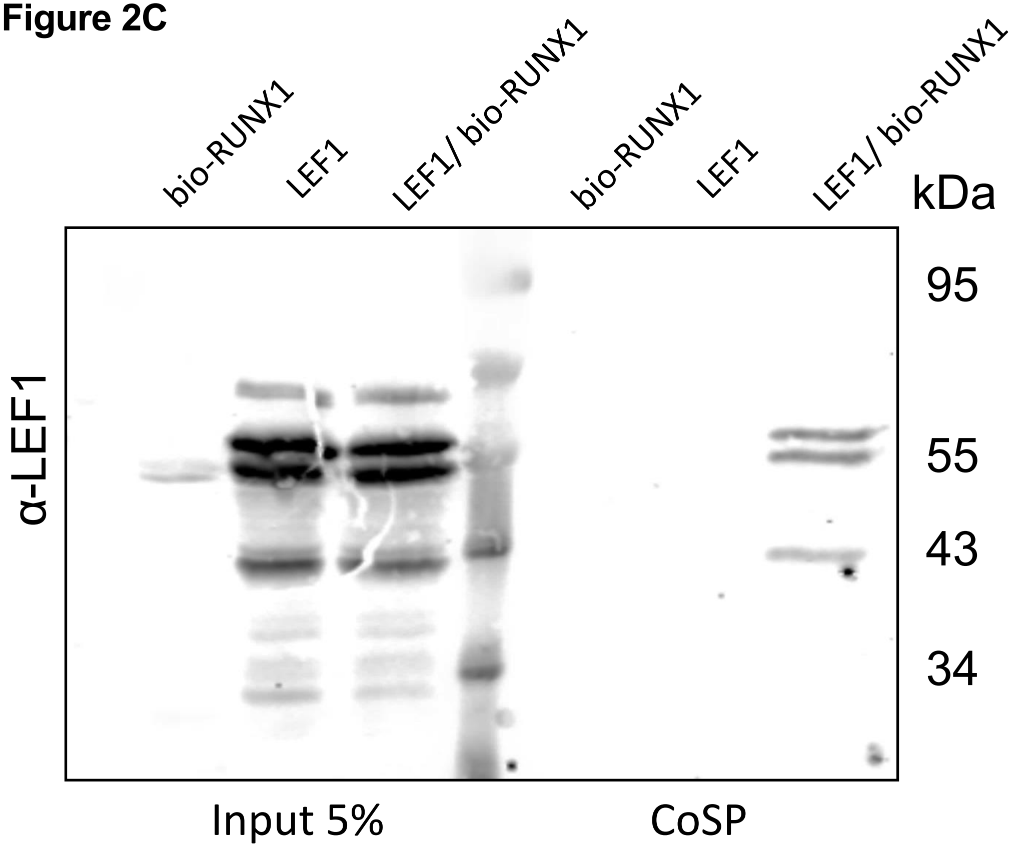

Related to Figure 2C

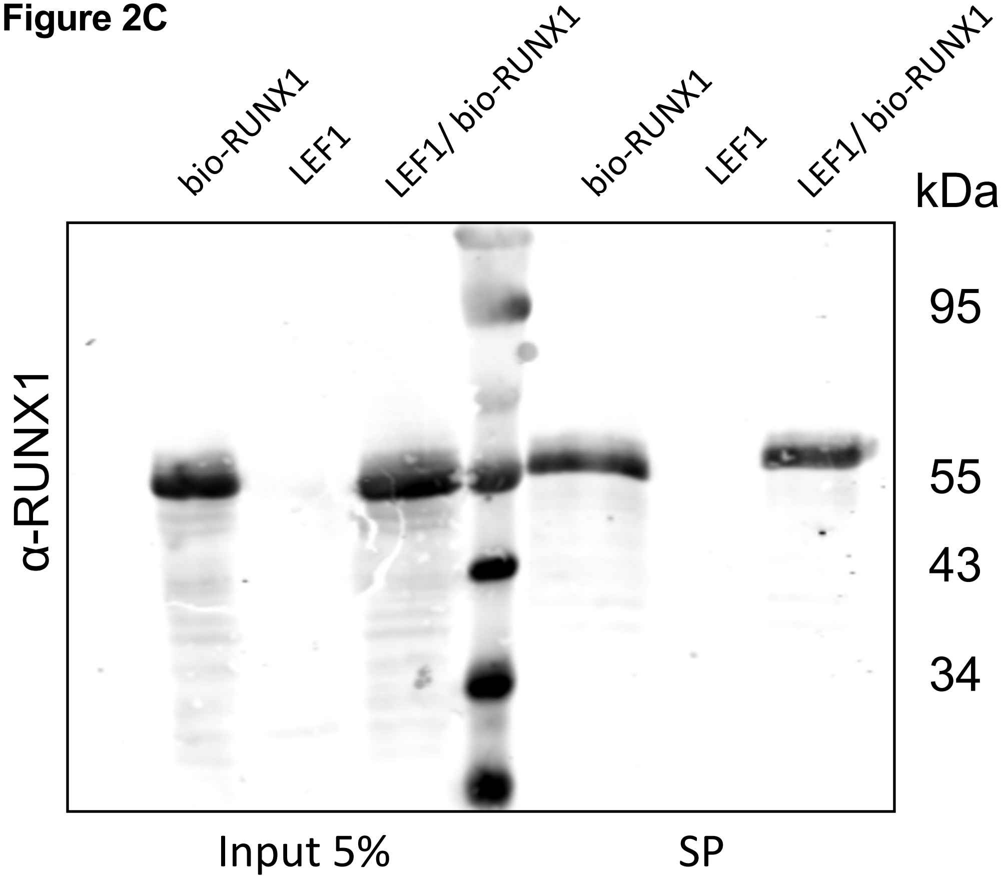

Related to Figure 2D

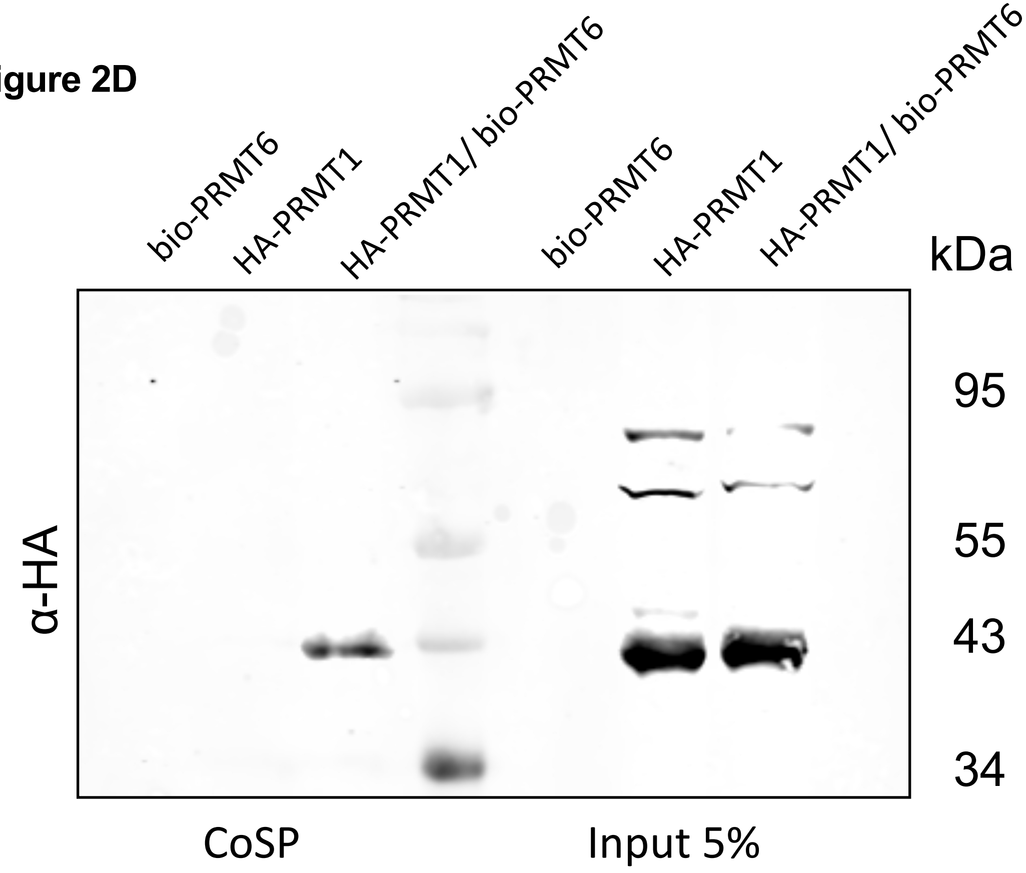

Related to Figure 2D

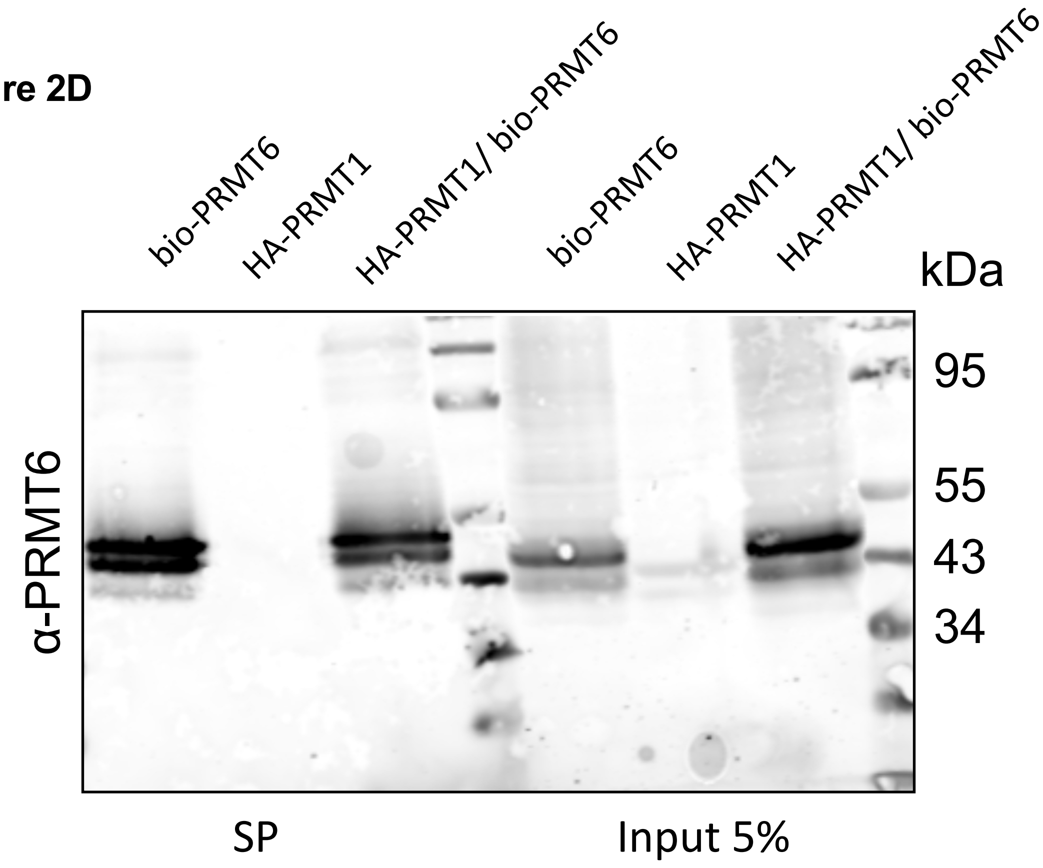

Fig 5A

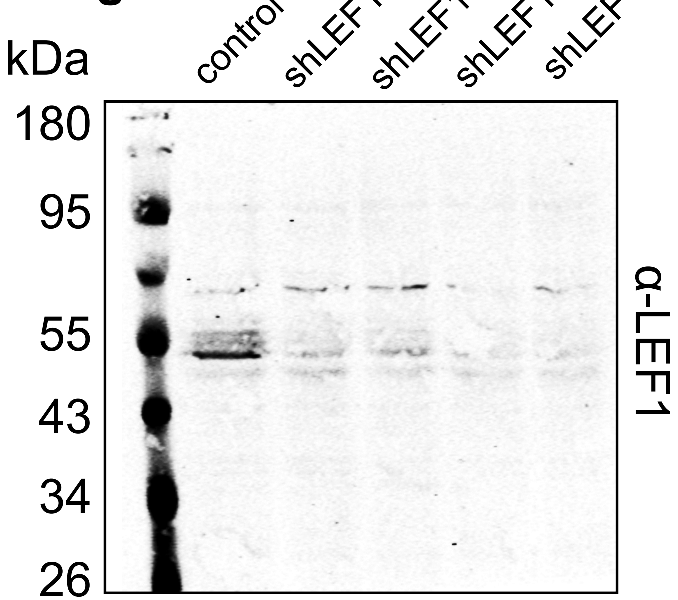

Fig 5A

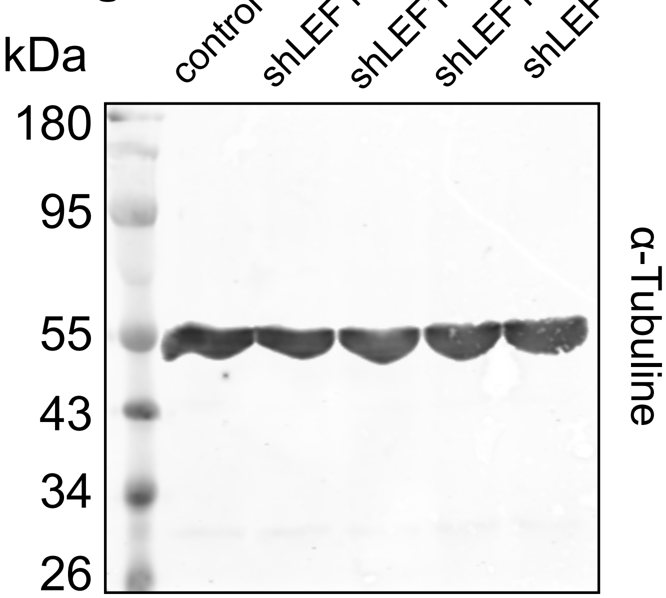

Fig 5B

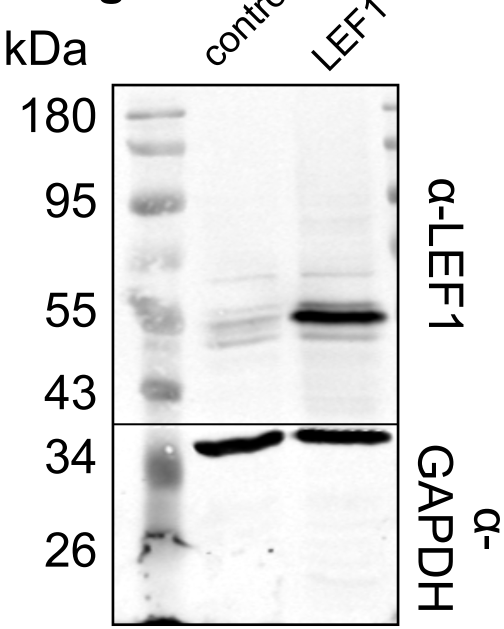

Related to Figure 5
